# Elucidating the role of tumor-associated ALOX5+ mast cells with transformative function in cervical cancer progression via single-cell RNA sequencing

**DOI:** 10.3389/fimmu.2024.1434450

**Published:** 2024-08-19

**Authors:** Fu Zhao, Junjie Hong, Guangyao Zhou, Tianjiao Huang, Zhiheng Lin, Yining Zhang, Leilei Liang, Huarong Tang

**Affiliations:** ^1^ Department of Gynecological Radiotherapy, Zhejiang Cancer Hospital, Hangzhou, China; ^2^ Shandong University of Traditional Chinese Medicine, Jinan, China; ^3^ Department of Gynecological Oncology, Zhejiang Cancer Hospital, Hangzhou, China; ^4^ Department of Lung Cancer, Tianjin Lung Cancer Center, National Clinical Research Center for Cancer, Key Laboratory of Cancer Prevention and Therapy, Tianjin’s Clinical Research Center for Cancer, Tianjin Medical University Cancer Institute and Hospital, Tianjin, China; ^5^ The First School of Clinical Medicine, Heilongjiang University of Traditional Chinese Medicine, Harbin, China; ^6^ Department of Clinical Laboratory, Zhejiang Cancer Hospital, Hangzhou, China

**Keywords:** single-cell RNA-sequencing, cervical cancer, tumor heterogeneity, prognosis, cancer immunotherapy

## Abstract

**Background:**

Cervical cancer (CC) is the fourth most common malignancy among women globally and serves as the main cause of cancer-related deaths among women in developing countries. The early symptoms of CC are often not apparent, with diagnoses typically made at advanced stages, which lead to poor clinical prognoses. In recent years, numerous studies have shown that there is a close relationship between mast cells (MCs) and tumor development. However, research on the role MCs played in CC is still very limited at that time. Thus, the study conducted a single-cell multi-omics analysis on human CC cells, aiming to explore the mechanisms by which MCs interact with the tumor microenvironment in CC. The goal was to provide a scientific basis for the prevention, diagnosis, and treatment of CC, with the hope of improving patients’ prognoses and quality of life.

**Method:**

The present study acquired single-cell RNA sequencing data from ten CC tumor samples in the ArrayExpress database. Slingshot and AUCcell were utilized to infer and assess the differentiation trajectory and cell plasticity of MCs subpopulations. Differential expression analysis of MCs subpopulations in CC was performed, employing Gene Ontology, gene set enrichment analysis, and gene set variation analysis. CellChat software package was applied to predict cell communication between MCs subpopulations and CC cells. Cellular functional experiments validated the functionality of TNFRSF12A in HeLa and Caski cell lines. Additionally, a risk scoring model was constructed to evaluate the differences in clinical features, prognosis, immune infiltration, immune checkpoint, and functional enrichment across various risk scores. Copy number variation levels were computed using inference of copy number variations.

**Result:**

The obtained 93,524 high-quality cells were classified into ten cell types, including T_NK cells, endothelial cells, fibroblasts, smooth muscle cells, epithelial cells, B cells, plasma cells, MCs, neutrophils, and myeloid cells. Furthermore, a total of 1,392 MCs were subdivided into seven subpopulations: C0 CTSG+ MCs, C1 CALR+ MCs, C2 ALOX5+ MCs, C3 ANXA2+ MCs, C4 MGP+ MCs, C5 IL32+ MCs, and C6 ADGRL4+ MCs. Notably, the C2 subpopulation showed close associations with tumor-related MCs, with Slingshot results indicating that C2 subpopulation resided at the intermediate-to-late stage of differentiation, potentially representing a crucial transition point in the benign-to-malignant transformation of CC. CNVscore and bulk analysis results further confirmed the transforming state of the C2 subpopulation. CellChat analysis revealed TNFRSF12A as a key receptor involved in the actions of C2 ALOX5+ MCs. Moreover, *in vitro* experiments indicated that downregulating the TNFRSF12A gene may partially inhibit the development of CC. Additionally, a prognosis model and immune infiltration analysis based on the marker genes of the C2 subpopulation provided valuable guidance for patient prognosis and clinical intervention strategies.

**Conclusions:**

We first identified the transformative tumor-associated MCs subpopulation C2 ALOX5+ MCs within CC, which was at a critical stage of tumor differentiation and impacted the progression of CC. *In vitro* experiments confirmed the inhibitory effect of knocking down the TNFRSF12A gene on the development of CC. The prognostic model constructed based on the C2 ALOX5+MCs subset demonstrated excellent predictive value. These findings offer a fresh perspective for clinical decision-making in CC.

## Introduction

Cervical cancer (CC) is among the most common malignancies, with the global statistics report for 2020 showing approximately 600,000 new cases of CC annually, leading to over 340,000 deaths. These figures place CC fourth in the incidence and mortality spectrum for women globally. More than 85% of these instances happen in countries with low and middle incomes, where the mortality rate is six times higher than in developed countries (1). The incidence and mortality rates of CC have declined in recent years because of enhanced early screening and wider administration of the HPV vaccine. However, there has been a rise in the incidence of CC among young women, indicating that it continues to be a significant public health concern (2). Furthermore, due to the atypical early symptoms of CC, most patients are diagnosed in advanced stages, posing significant challenges to treatment.

The primary method of treating locally advanced CC according to the 2024 NCCN recommendations is concurrent chemoradiotherapy (CCRT). Nevertheless, recurrence or metastasis affects about 50% of individuals following therapy (3). Research has indicated that the likelihood of cancer returning in patients with locally advanced CC stages IB-IIB after CCRT is between 10% and 20%, however it increases to 50% to 70% for stages IIB-IVA (4). In addition, the use of carboplatin and paclitaxel as adjuvant chemotherapy after radiation does not result in a substantial increase in overall survival (OS) or progression-free survival (5).

The progress in cancer treatment has been significant due to advancements in tumor immunology, immunotherapy, and molecular targeted therapies. Immune checkpoint blockade (ICB) therapy has been used to treat several solid cancers, such as lung cancer and melanoma, by targeting important molecules such CTLA-4, PD-L1, and PD-1. In addition, ICB therapy shows promising potential in cases of recurrent or metastatic CC. Studies suggest that ICB monotherapy increases OS by 3.5 months compared to chemotherapy alone. Furthermore, when ICB is combined with chemotherapy, with or without anti-angiogenic treatment, it can extend OS by almost one year (6). In addition, molecular targeted therapeutics are being investigated in the context of CC. *In vitro* studies have confirmed that pathways such as VEGF, EGFR/HER2, and PI3K/AKT/mTOR are strongly linked to a negative prognosis in CC patients (7). However, apart from Bevacizumab, the outcomes of phase II trials for other targeted therapies have not been encouraging, failing to progress to phase III trials (8). Moreover, immunotherapy and molecular targeted therapies struggle to sustain long-term efficacy in clinical settings due to tumor heterogeneity and the onset of primary or acquired drug resistance. Indeed, data indicate that more than 50% of patients initially responsive to ICB therapy exhibit disease progression within two years (9). Consequently, despite advances in immunotherapy, the treatment and survival outcomes for CC patients continue to be worrisome, highlighting the need for new immunotherapeutic strategies.

Mast cells (MCs), widely distribute across all tissues, are known to secrete a plethora of vasoactive mediators and pro-inflammatory factors (10). MCs, which are a component of the innate immune system, play an important part in the manifestation of chronic inflammatory disorders that are linked to cancer. Furthermore, they are an essential component of the inflammatory milieu that controls the genesis and progression of tumors. Studies investigating the function of MCs in cancer have shown varied results: commonly, MCs appear to facilitate tumor cell growth, often enhancing the progression of cancers, including thyroid, gastric, and lung cancers. Nevertheless, in cases such as breast cancer, MCs have the ability to stimulate the attraction of immune cells, which might potentially have an anti-tumor effect (11). Furthermore, MCs may play a non-contributory role in tumors such as renal cell carcinoma, potentially acting as mere inert bystanders (12). Similarly, early studies investigating the association between MCs and CC have yielded contradictory results. Graham et al. observed a decrease in MC count with tumor progression (13) whereas another study found no significant difference in the number of MCs between grades I-III of cervical intraepithelial neoplasia, but a notable increase in MCs numbers in infiltrating CC suggested that MCs played a role in promoting the progression and dissemination of tumor around and within the cervix (14). Consequently, it is imperative to investigate the interactive mechanisms between CC and MCs.

In recent years, single-cell sequencing technology has come to be as a burgeoning technique, enabling multifaceted analysis at the single-cell resolution of the genome, proteome, epigenome, and spatial transcriptome (15–18). By elucidating the features, developmental trajectories, and underlying mechanisms of distinct cellular subgroups, it has furnished novel insights into the realm of tumor biology, facilitating the refinement of therapeutic strategies and propelling the progress of personalized medicine. The composition of CC tissue represents a complex ecosystem comprising diverse cellular subgroups, including immune cells, EPCs, and MCs (19). The tumor microenvironment (TME) and tumor heterogeneity have significant impacts on the onset (20), advancement and prognosis of CC. However, the full extent of MCs heterogeneity within the TME of CC remains incompletely elucidated (21). We thus seek to investigate the cellular heterogeneity within the tumor and expose its complex cellular states by using single-cell RNA sequencing (scRNA-seq) analysis on a CC dataset derived from the ArrayExpress database. It is our aspiration to offer fresh perspectives on the diagnosis, management, and prognosis of CC to improve patient outcomes and raise survival rates.

## Method

### Acquisition of single-cell data

The single-cell data for CC was acquired from the ArrayExpress database (https://www.ebi.ac.uk/arrayexpress/), under the dataset accession number E-MTAB-12305 (22). Bulk RNA sequencing data for CC was acquired from of the University of California Santa Cruz (UCSC, https://xena.ucsc.edu/) Xena. As we utilized publicly available database information in our study, ethical approval was not required.

### Filtering and processing of the raw data

To analyze scRNA-seq data, we utilized R software (4.2.0) along with the “Seurat” software package (4.3.0) (23). To enhance the accuracy and reliability of the scRNA-seq data, we utilized the “DoubletFinder” software package (version 2.0.3) (24) for quality control, detection, and filtration of probable low-quality and aberrant cells (25, 26). The nFeature parameter must have a value within the range of 300 to 6000, whereas the nCount parameter must have a value within the range of 500 to 100,000. The proportion of genes related to red blood cells in the cell was less than 5% of the total number of genes. Furthermore, cells with mitochondrial gene expression exceeding 25% of the overall expression were excluded.

In order to analyze the filtered samples, we utilized the Seurat package’s “NormalizeData” and “FindVariableFeatures” functions to normalize the data and identify the top 2000 genes with high variability (27–29). Afterwards, we utilized the “ScaleData” function to normalize the analyzed data and then addressed batch discrepancies among datasets by employing principal component analysis with the harmony R package (version 0.1.1) (30–32). Ultimately, we conducted dimensionality reduction and clustering using the most important 30 principal components.

The analysis of copy number variation (CNV) in scRNA-seq data was conducted using the inferCNV R package (version 1.6.0) obtained from the GitHub repository of the Broad Institute (https://github.com/broadinstitute/inferCNV). This software package enables the distinction between cancerous and healthy cells by analyzing the chromosomal locations and gene expression levels to determine copy number variations. Cells with high CNV scores were defined as Tumor-EPCs.

### The identification of differentially expressed genes (DEGs) and cell types

We utilized the “FindClusters” and “FindNeighbors” functions in Seurat to carry out cell clustering (33). We used the Seurat function “FindAllMarkers” to detect DEGs in each cluster. Most of the identified marker genes for cell clusters were obtained from the CellMarker (http://xteam.xbio.top/CellMarker/), in addition to some citations from past research. Cell annotation was conducted through manual curation. Afterward, we utilized the UMAP technique to visualize the data.

### Slingshot pseudotemporal analysis

Version 2.6.0 of the Slingshot software program was used to infer the cell lineage during the differentiation of the MCs subpopulations (34). The function “getLineages” was utilized to calculate the levels of cellular expression for every lineage.

### Cellular stemness analysis

In order to assess the scores of gene sets in single-cell transcriptomic data, we employed the AUCell method. We utilized the AUCell package and employed the “AUCell_buildRankings” function to rank the stemness gene set based on the magnitude of scores.

### Functional enrichment analysis

We conducted a functional analysis using the ClusterProfiler R software package based on Gene Ontology (GO) analysis and Kyoto Encyclopedia of Genes and Genomes (KEGG) (35–38). In order to perform gene set enrichment analysis (GSEA), we took into account the collective gene expression patterns within the gene sets. For this research, we employed the Molecular Signatures Database (MSigDB, https://www.gsea-msigdb.org/gsea/msigdb) to identify pathways that showed a significant enrichment (39–41).

### Cell communication

The CellChat R package (version 1.6.1) (42) was utilized for quantitatively inferring and analyzing cellular interactions from scRNA-seq data. The “netVisual_diffInteraction” function was used to analyze variations in the strength of intercellular communication, while the “identifyCommunicationPatterns” function was utilized to determine the quantity of communication patterns. Scatter plots, heatmaps, and various visualization techniques were utilized to analyze the signals coming in and out of every cell visually (43).

The CellChat database (http://www.cellchat.org/) was subsequently utilized to identify signaling pathways and receptor pairings associated with specific types of MCs that are relevant to cancer. The “netVisual_bubble” function was employed to assess the probability of communication between ligand-receptor pairings regulated by distinct cell clusters and those originating from dissimilar cell clusters.

### Development and validation of the prognostic prediction model

First, we filtered the most important prognostic genes using univariable Cox analysis and least absolute shrinkage and selection operator (LASSO) regression analysis (44–46). We next computed the hazard coefficients for every prognostic gene by multivariable Cox regression analysis (47–50). This enabled us to establish a risk scoring model (Risk score = $\sum_{\text{i}}^{\text{n}}\text{Xi}\times \text{Yi}$ , where X represents the coefficient and Y represents the gene expression level) (51–53). On the basis of the optimal cutoff values that were determined by the “surv_cutpoint” function, we organized the data into groups. We analyzed the predictive results for various groups of patients by performing survival analysis on the risk scoring model we developed with the R package ‘Survival’ (version 3.3.1) and displaying the survival curves with the “ggsurvplot” function (54, 55). By plotting receiver operating characteristic (ROC) curves with the “timeROC” package (version 0.4.0), we evaluated the predictive model’s accuracy (37, 56–59).

In addition, we performed a multivariable Cox regression analysis to validate the independent predictive value of the risk score. Furthermore, we created a nomogram to predict the OS at 1, 3, and 5 years. The accuracy of the nomogram’s predictions was verified by the utilization of the C-index and calibration curves.

### Immune microenvironment analysis

We utilized the CIBERSORT R package (version 0.1.0) to calculate immune-related scores for 22 immune cell types (60–62). Afterward, we utilized three different tools “CIBERSORT”, “ESTIMATE”, and “Xcell” to thoroughly assess the immune surroundings of the patients (63, 64). Additionally, we analyzed variations in levels of immune cell infiltration and the expression of genes related to immune checkpoints. We next ran correlation studies between OS, risk scores, immune cells, and model genes (65). We also evaluated the response to tumor immune therapy using Tumor Immune Dysfunction and Exclusion (TIDE, http://tide.dfci.harvard.edu) program.

### Cell lines and cell culture

The HeLa and Caski cell lines were obtained from the Cell Resource Center at the Shanghai Institute for Biological Sciences, which is part of the Chinese Academy of Sciences. The cells were cultured individually in RPMI 1640 media supplemented with 10% fetal bovine serum (FBS) (Gibco BRL, USA), and 1% penicillin-streptomycin. The cell lines were cultivated under conventional conditions, with a temperature of 37°C and a 5% CO_2_ atmosphere.

### siRNA knockdown

RNA constructs (GenePharma, Suzhou, China) helped to knockdown TNFRSF12A. On a 6-well plate set at a 50% density, the cells were planted. They then underwent knockdown (si-TNFRSF12A-1 and si-TNFRSF12A-2) and negative control (si-NC) transfecting. Lipofectamine 3000RNAiMAX (Invitrogen, USA) was used for transfection under manufacturer directions. Every si-RNA (RIbbio, China) was transfected into cells. **Supplementary Table S1** shows the siRNA sequence from 5’ to 3’.

### Cell viability assay

Transfected cell viability was assessed with the Cell Counting Kit-8 (CCK-8, A311-01, Vazyme) (66). The suspension of cells was placed in a 96-well dish with 5×10^3^ cells in each well and left to incubate for 24 hours. Afterward, 10 microliters of CCK-8 labeling reagent were added to every well, followed by incubation of the plate at 37 degrees Celsius in a light-shielded setting for a duration of 2 hours. Cell viability was evaluated by measuring the absorbance at 450 nm over a period of four days. Mean optical density values were determined and graphically represented using a line graph.

### Quantitative polymerase chain reaction (qPCR)

RNA extraction was performed using the Trizol reagent, and reverse transcription was carried out using the PrimeScript™ Kit. The qPCR reaction was conducted using SYBR Green premix (67). The primer sequences used were listed in **Supplementary Table S1**.

### Wound-healing assay

The cells that had been successfully transfected with stable genetic material were placed in a 6-well plate and grown in a controlled environment within a cell culture incubator until they reached full coverage of the plate. With a sterile 200 μL plastic pipette tip, the cells in each culture well were delicately scraped and then rinsed with PBS to eliminate any cell debris. Afterwards, the cells were placed in a culture medium without serum and incubated. Photographs of the scratch injuries were taken at 0 hours and 48 hours, and the width of the scratches was quantified using the Image-J software. The wound healing percentage was determined by applying the formula: (the scratch area in 0-48 hours × 100)/the area in 0 hours.

### Transwell assays

The cell migration capacity was evaluated using a Transwell test. The top compartment of a 24-well plate was covered with a matrix gel solution (BD Biosciences, USA), and the cell mixture was placed in the top compartment, while a culture medium containing 10% FBS was added to the bottom compartment. The plates were subsequently placed in a cell culture incubator and kept there for a duration of 48 hours. Following the removal of cells from the upper chamber, the surviving cells on the lower surface were treated with 4% paraformaldehyde for fixation and then stained with 0.1% crystal violet (Solarbio, China). The cells in five randomly selected fields of vision were quantified using an optical microscope.

### 5-Ethynyl-2’-deoxyuridine proliferation experiments

The HeLa and Caski cell lines that were transfected were placed into a 6-well cell culture plate with 5×10^3^ cells in each well. Following a 24-hour incubation period at ambient temperature, the EdU working solution was introduced into the cell culture medium and left to incubate for 2 hours. Afterwards, the cells were rinsed twice with PBS and then treated with a 4% paraformaldehyde solution for 15 minutes to immobilize them. Next, the cells were subjected to treatment with glycine at a concentration of 2 mg/ml and 0.5% Triton X-100 for a duration of 15 minutes. Ultimately, the cells were subjected to a treatment involving the addition of 1 ml of 1X Apollo and 1 ml of 1X Hoechst staining reaction solution, which lasted for a duration of 30 minutes. Cell proliferation was assessed by capturing images using a fluorescence microscope.

### Statistical analysis

We performed statistical analysis using the R software (version 4.2.0). The statistical significance of the data was determined by calculating the p-values. The levels of significance were marked with asterisks: *P < 0.05, **P < 0.01, ***P < 0.001, and ****P < 0.0001. "ns" was used to say that there was no significant difference.

## Result

### Single-cell analysis of the primary cell types in CC

**Figure 1:**
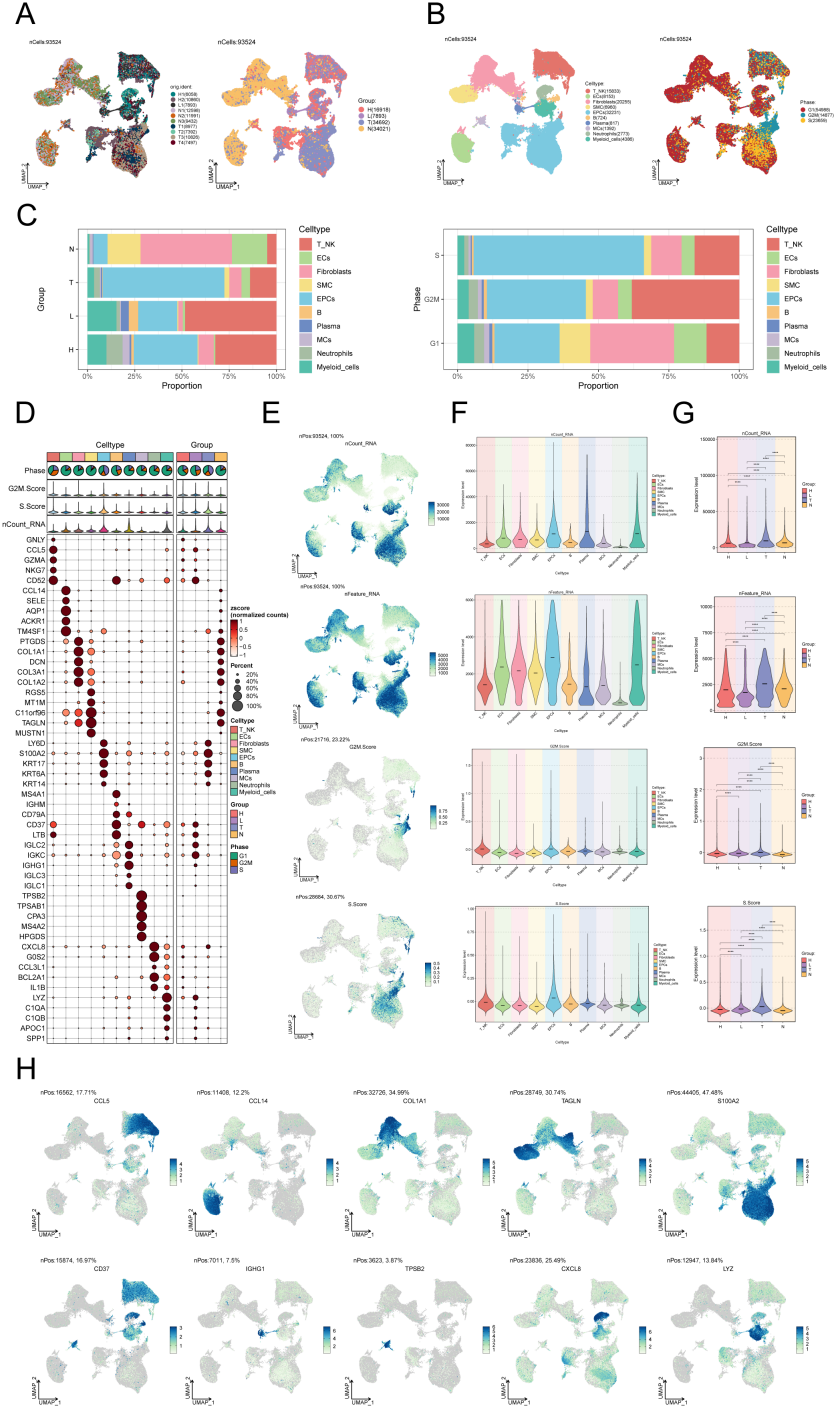
Single-cell landscape of CC. **(A)** The UMAP plots of the single cell spectrum depicted in this paper is presented. The plots exhibited distinguished coloration based on the sample source (on the left) and tissue type (on the right). **(B)** UMAP plot on the left annotated cell types (T_NK cells, ECs, SMCs, EPCs, B cells, plasma cells, MCs, neutrophils, and myeloid cells) based on known lineage-specific marker genes (represented by colors). On the right, the UMAP plot depicted the distribution of cells in different cell cycle phases (G1, G2M, S). **(C)** Bar graphs depicted the relative proportions of the ten distinct cell types across various tissue types (left) and cell cycle (right). **(D)** Bubble plot visually represented the expression levels of diverse marker genes according to annotated cell types. The coloration of the bubbles is determined by normalized data, while the size of the bubbles denotes the proportion of gene expression. **(E)** UMAP plots illustrated the distribution of nCount_RNA, nFeature_RNA, G2M.score, and S.score across ten distinct cell types within CC. **(F, G)** Violin plots depicted the expression levels of nCount_RNA, nFeature_RNA, G2M.score, and S.score across ten cell types **(F)** as well as various tissue types **(G)** in the context of CC. . ****P < 0.0001. **(H)** UMAP plots showed DEGs across distinct cell types in the context of CC.

We performed scRNA-seq analysis on 10 CC samples to explore the heterogeneity of cell types. Following quality assurance, a combined 93,524 cells were collected from 2 High Squamous Intraepithelial Lesion (H) specimens, 1 Metastatic Lymph Node (L) specimen, 4 Cervical Tumors (T) specimens, and 3 Normal Cervix (N) specimens. After batch effect removal, UMAP dimensionality reduction clustering was applied to high-quality cells to visualize distinct groupings (H: High Squamous Intraepithelial Lesion, L: Metastatic Lymph Node, T: Cervical Tumors, N: Normal Cervix) (**Figure 1A**). Using known marker genes for typical cell types, we annotated the 93,524 high-quality cells, resulting in 10 cell types: T_NK cells, endothelial cells (ECs), fibroblasts, smooth muscle cells (SMCs), epithelial cells (EPCs), B cells, plasma cells, MCs, neutrophils, and myeloid cells. The cell cycle distribution differences among these cell types were shown (**Figure 1B**). Our observation of MC infiltration in CC tissues primarily consisted of H and N clusters, leading us to hypothesize that MCs could be involved in the conversion of tumoral epithelium. The bar graph on the left illustrates the relative proportions of the ten distinct cell types across different tissue types (H, L, T, N), while the bar graph on the right showcases the relative proportions of different cell types at various stages of the cell cycle (**Figure 1C**).

In addition, an expression bubble plot was utilized to depict the expression levels of the top 5 marker genes for each cell type (**Figure 1D**). By examining the distribution patterns and levels of expression of nCount_RNA, nFeature_RNA, S.score, and G2M.score in various cell types, we may gain a deeper understanding of the differences between these cell types (**Figures 1E, F**). The violin plots demonstrate that the tumor group displays elevated amounts of nCount_RNA, nFeature_RNA, S.score, and G2M.score, suggesting a heightened cellular proliferation within this group (**Figure 1G**). The DEGs across the 10 cell types are illustrated in the UMAP plots (**Figure 1H**).

### Visualization of MCs subpopulations in CC

**Figure 2:**
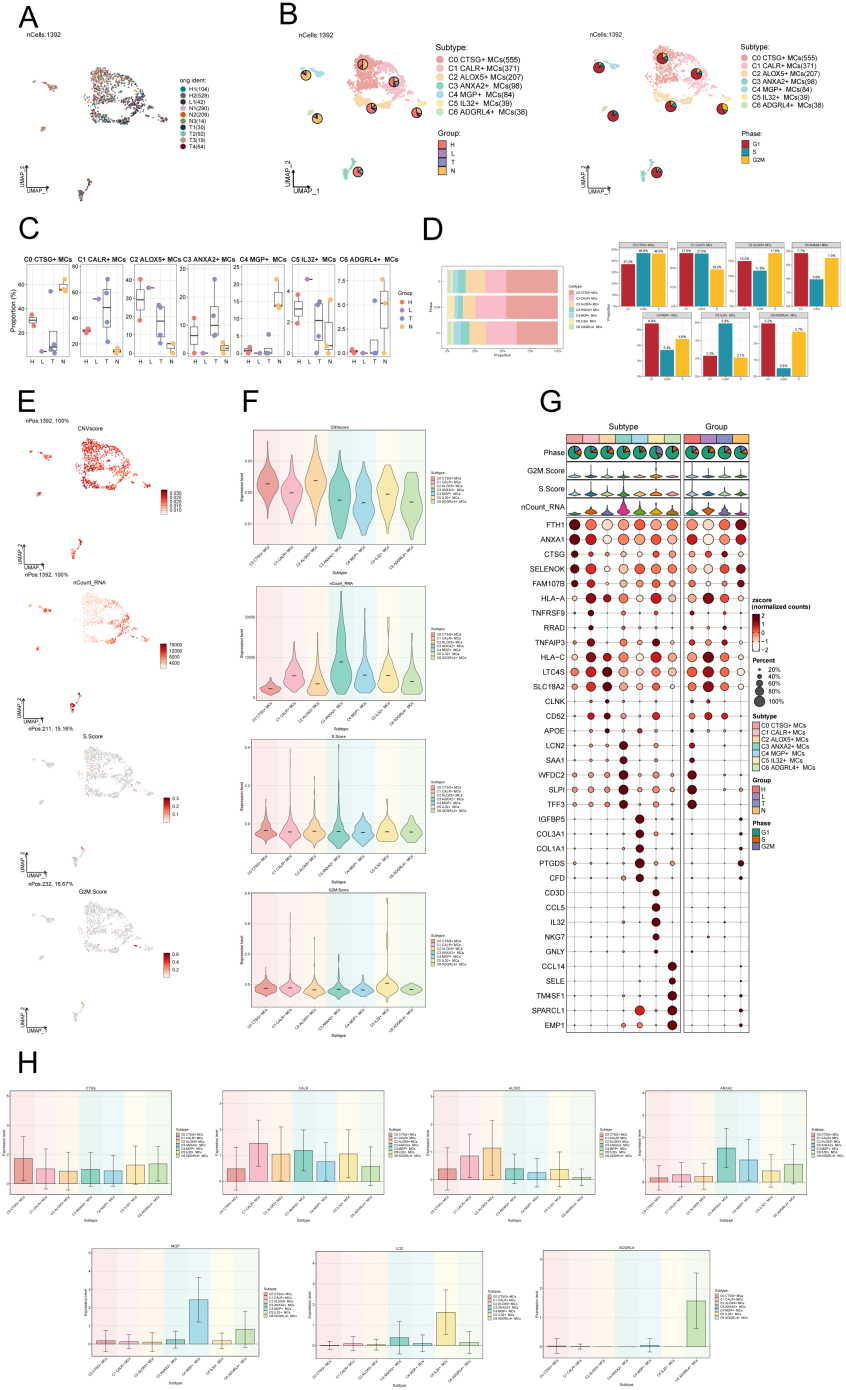
Visualization of MCs subpopulations in CC. **(A)** UMAP plot demonstrated the origin of the samples and the clustering of 1392 high-quality cells using the downscaling technique through the Seurat method. **(B)** The cells were annotated according to recognized lineage-specific marker genes (indicated by color): C0 represented CTSG+ MCs, C1 represented CALR+ MCs, C2 represented ALOX5+ MCs, C3 represented ANXA2+ MCs, C4 represented MGP+ MCs, C5 represented IL32+ MCs, and C6 represented ADGRL4+ MCs. A pie chart was employed to illustrate the MCs subpopulation in terms of tissue type and cell cycle. On the left side, the groups (H, L, T, N) were specified, while on the right side, the phases (G1, S, G2M) were delineated. **(C)** Box plots depicted the distribution of different tissue types among various subtypes of MCs. **(D)** Bar graph displayed the varying cell cycle occupancies of the seven cell subpopulations of MCs in CC. **(E)** UMAP plots exhibited the distribution of CNVscore, nFeature_RNA, S.score, and G2M.score across MCs subpopulations. **(F)** Violin plots demonstrated the expression levels of CNVscore, nFeature_RNA, S.score, and G2M.score across MCs subpopulations. **(G)** Bubble plot exemplified the differential expression of top5maker genes within MCs subpopulations and across distinct tissue types. The coloration of the bubbles signifies the level of gene expression, while the size reflects the proportionate percentage of gene expression within the subpopulations. **(H)** Bar graphs illustrated the expression levels of marker genes within each subgroup.

After performing dimensionality reduction and clustering, a total of 1392 CC-associated MCs were obtained. The UMAP plot illustrated the origins of the 10 samples and the removal of batch effects in CC cells (**Figure 2A**). We identified seven distinct subgroups of MCs and annotated them based on their respective cell marker genes: C0 CTSG+ MCs (555), C1 CALR+ MCs (371), C2 ALOX5+ MCs (207), C3 ANXA2+ MCs (98), C4 MGP+ MCs (84), C5 IL32+ MCs (39), and C6 ADGRL4+ MCs (38). Using the UMAP plot combined with pie charts depicting cell proportions, we showcased the distribution of these seven MCs subgroups across different groups (H, L, T, N) and cell cycle phases (G1, S, G2M) (**Figure 2B**). Among them, subgroups C0, C4 and C6 predominantly originated from normal tissues, while subgroups C1, C2, C3 and C5 had a higher proportion of tumor tissue representation. Subsequently, we utilized box plots to display the distribution of MCs from different tissue types within each subgroup (**Figure 2C**). The results revealed that Normal Cervix mainly clustered in subgroups C0, C4, and C6, while Cervical Tumors were predominantly concentrated in subgroup C1, with some presence in subgroups C2 and C3. High Squamous Intraepithelial cells were primarily distributed in subgroups C0, C1, and C2, with a smaller portion found in subgroup C3. A bar graph was employed to demonstrate the proportions of cell cycle phases across the different MCs subgroups of CC (**Figure 2D**), indicating no significant differences among the seven subgroups in terms of cell cycle distribution (G1, S, G2M).

We utilized inferCNV to identify chromosomal CNVs within cells, aiming to investigate the malignancy level of tumors and abnormal states of cells (68). This approach assists in distinguishing tumor cells from normal cells and identifying clusters of abnormal cells within tumor cells. The heatmap displays the CNV profiles of EPCs inferred using ECs as a reference (**Supplementary Figure S1A**). The results indicate the presence of abnormal chromosomal copy number amplifications or deletions in malignant EPCs of CC. In **Supplementary Figure S1B**, the inferred CNVs of each cell subpopulation are illustrated. Next, we employed UMAP plots to visualize the CNV scores, nCount_RNA, S.score, and G2M.score of the MCs subgroups. The results were presented using violin plots (**Figures 2E, F**). The C2 ALOX5+ MCs subgroup displayed the highest CNV score, indicating a greater occurrence of copy number variants in comparison to other subgroups. This suggests a possibly higher level of malignancy. On the other hand, the C3 ANXA2+ MCs subgroup displayed a higher nCount_RNA score, suggesting a relatively active cellular proliferation state. In **Figure 2G**, the top 5 marker genes’ differential expression was highlighted within the MCs subgroups. The findings showed that the leading 5 marker genes in the C2 ALOX5+ MCs subgroup were similarly present in other MCs subgroups.

We ultimately employed bar graphs to exhibit the levels of marker genes expression in different subcategories (**Figure 2H**).

### Slingshot analysis of proposed temporal trajectories of MCs subpopulations

**Figure 3:**
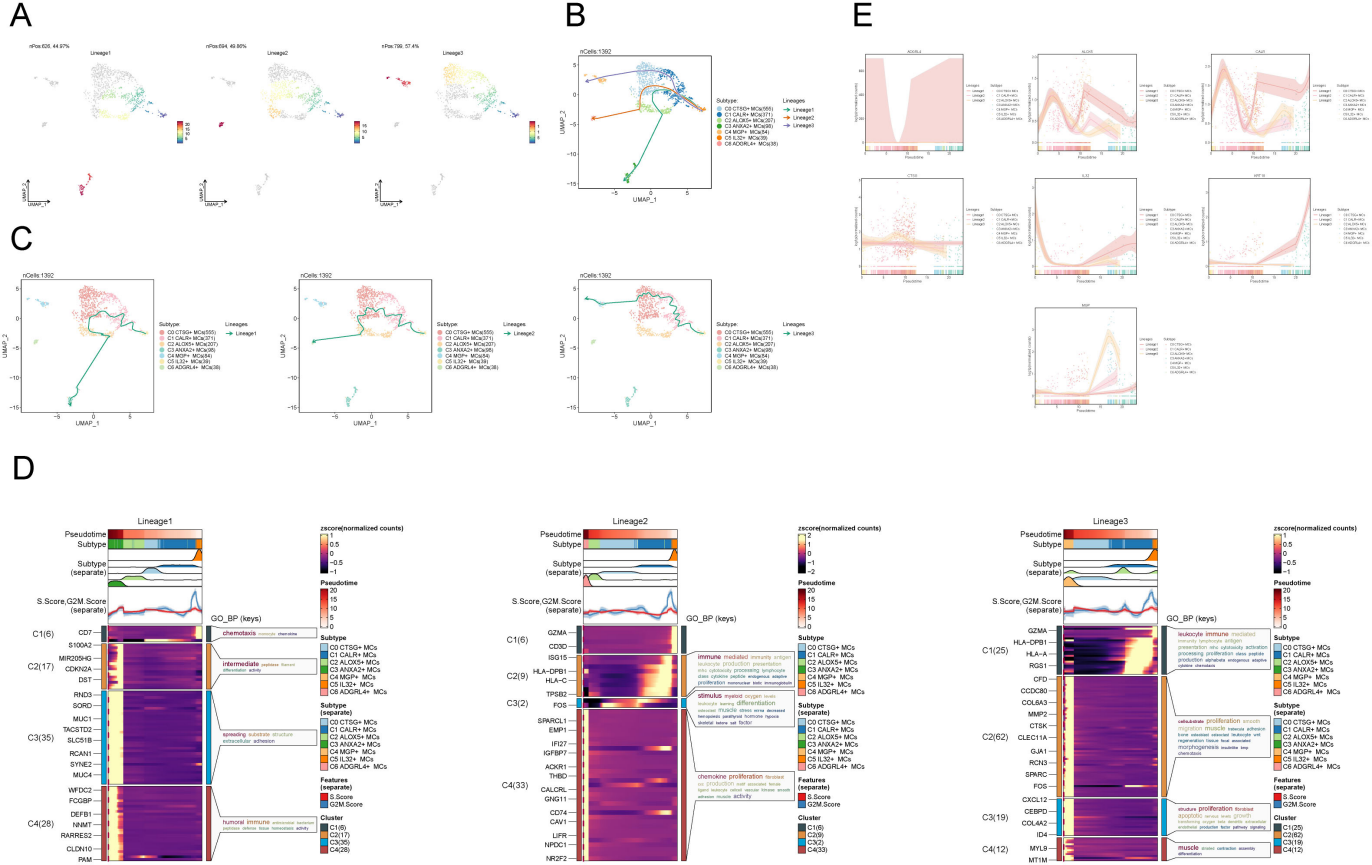
Slingshot analysis of proposed temporal trajectories of MCs subpopulations. **(A)** UMAP plots demonstrated the distribution of differentiation trajectories of MCs, fitted by slingshot, across the entire MCs population. Lineage1 nPos:626,44.97%, Lineage2 nPos:694,49.86%, Lineage3 nPos:799,57.4%. **(B, C)** UMAP plots showed the distribution of the three pseudotemporal trajectories of MCs in all MCs clusters. Solid lines indicate differentiation trajectories with arrows pointing to the direction of differentiation (from naive to mature). Lineage1: C5→C1→C0→C2→C3; Lineage2:C5→C1→C0→C2→C6; Lineage3:C5→C1→C0→C4. **(D)** The results of the GO-BP enrichment analysis confirmed the biological processes corresponding to the three pseudotemporal trajectories of MCs subpopulations. **(E)** Kinetic trend plot showcased the fluctuation and dispersion of marker gene expression in the MCs subpopulations along the three differentiation trajectories in pseudotime. The plot was color-coded according to cell type.

To infer the lineage trajectory and pseudotime sequence of MCs, we employed slingshot analysis to assess the distribution of MCs differentiation trajectories across all MCs, visually represented through UMAP plots (**Figure 3A**). We found 3 cell lineage trajectories of the MCs subpopulations (**Figures 3B, C**). Lineage 1 followed the path C5 → C1 → C0 → C2 → C3; Lineage 2 followed the path C5 → C1 → C0 → C2 → C6; Lineage 3 followed the path C5 → C1 → C0 → C4. Slingshot analysis revealed that the differences among the three trajectories mainly reside in the middle to late stages. Combined with the analysis results depicted in **Figure 2C**, the C3 subpopulation was positioned at the end of Lineage1, predominantly present in CCs. On the other hand, although the C4 and C6 subpopulations were located at the ends of lineage3 and lineage2, respectively, they exhibited a significantly higher proportion in the normal cervix. Therefore, we inferred that lineage1 represents the differentiation line of MCs associated with the tumor. Moreover, we noted that both lineage1 and lineage2 pass through the C2 subpopulation at the late stage of differentiation, but with different endpoints. From this observation, we speculated that the C2 subpopulation likely plays a crucial role in the differentiation of tumor-associated MCs. Subsequently, Gene Ontology Biological Process (GO-BP) enrichment analysis was employed to validate the biological processes associated with the three lineage paths of MCs subpopulations (**Figure 3D**). The enrichment results indicated the following: C1: leukocyte immune, mediated immunity and lymphocyte antigen presentation; C2: cell-substrate, muscle; C3: smooth, proliferation; C4: humoral, tight, junction. The dynamic trends plot depicted the changes in expression levels and distribution patterns of marker genes for various subpopulations of MCs over three differentiation trajectories in pseudotime (**Figure 3E**).

### Expression of stemness gene sets in MCs subpopulations

**Figure 4:**
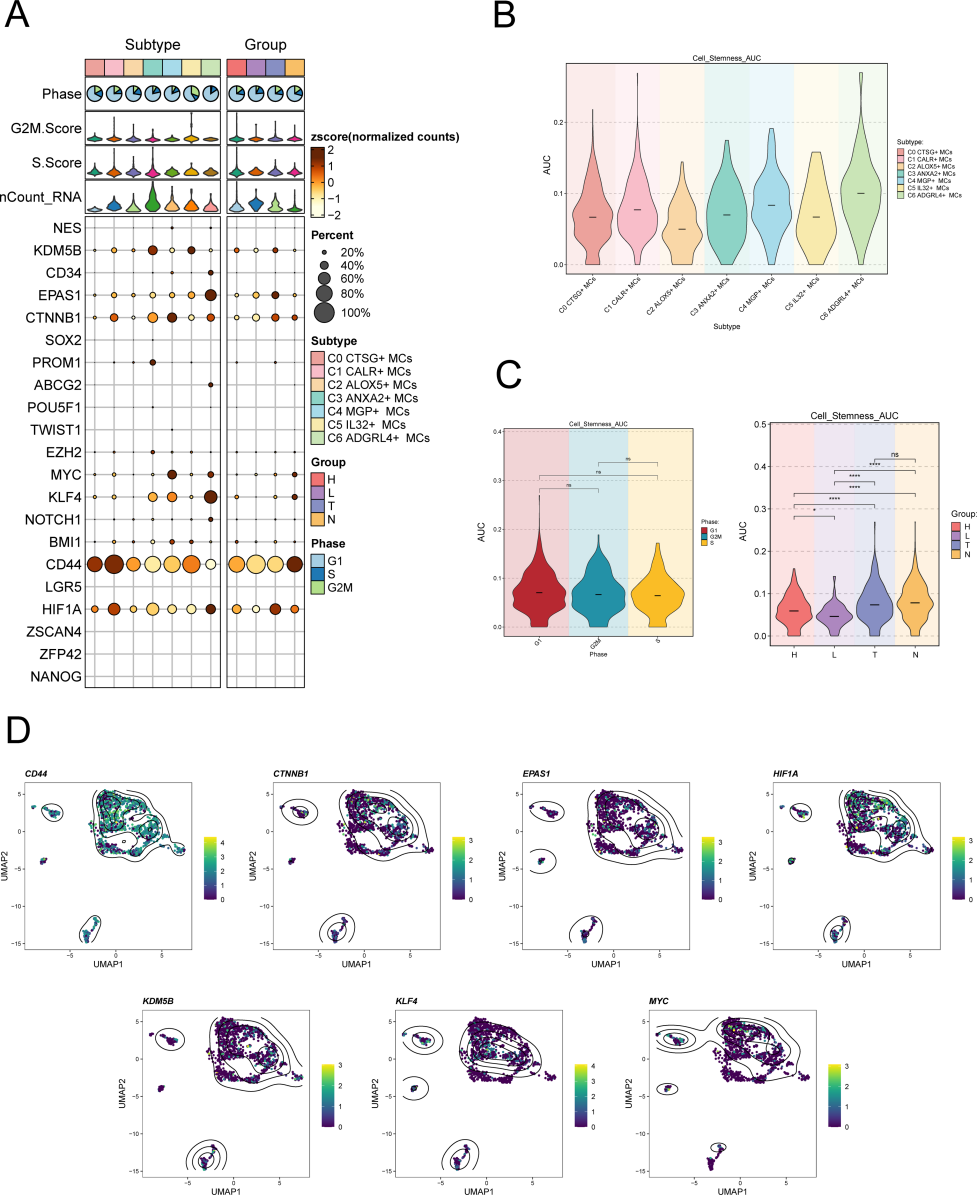
Expression of stemness gene sets in MCs subpopulations. **(A)** Bubble plot demonstrated the differential expression of stemness genes across various MCs subpopulations and tissue types. The size of the bubbles indicates gene expression score and the color represents the normalized data. **(B, C)** Violin plots demonstrated the AUC value of stemness genes in different MCs subpopulations **(B)**, cell cycle and tissue types **(C)**. *P < 0.05, and ****P < 0.0001 indicated a significant difference and "ns" indicated a non-significant difference. **(D)** UMAP plots showed the spatial arrangement of stemness genes among various subtypes of MCs, presented through the visualization of contour density.

To examine the expression of stemness genes in distinct subgroups of MCs and comprehend their ability to differentiate, we employed a bubble plot to visually represent the variation in expression of stem cell genes across these subgroups. The results demonstrated the expression of stem cell genes CD44, CTNNB1, EPAS1, HIF1A, KDM5B, KLF4, and HIF1A in distinct tissue types and subpopulations of MCs, as shown in **Figure 4A**. Subsequently, we undertook further analysis to assess the variations in cellular stemness among different subpopulations (**Figure 4B**). The results demonstrated that C2 ALOX5+MCs exhibit a lower level of cell stemness, suggesting a higher degree of differentiation. The violin displayed the variations of cellular stemness across different cell cycles and tissue types (**Figure 4C**). The above findings suggested that the Normal Cervix tissue exhibits the highest level of cell stemness. Consequently, we could deduce that the remaining three tissue types may have undergo differentiation that originated from the Normal Cervix. Moreover, there was no significant disparity in cellular stemness between different cell cycles. Finally, the stemness genes with relatively elevated expression levels in **Figure 3A** were showcased in all MCs through UMAP plots and contour plots (**Figure 4D**).

### Enrichment analysis of MCs subpopulations in CC

**Figure 5:**
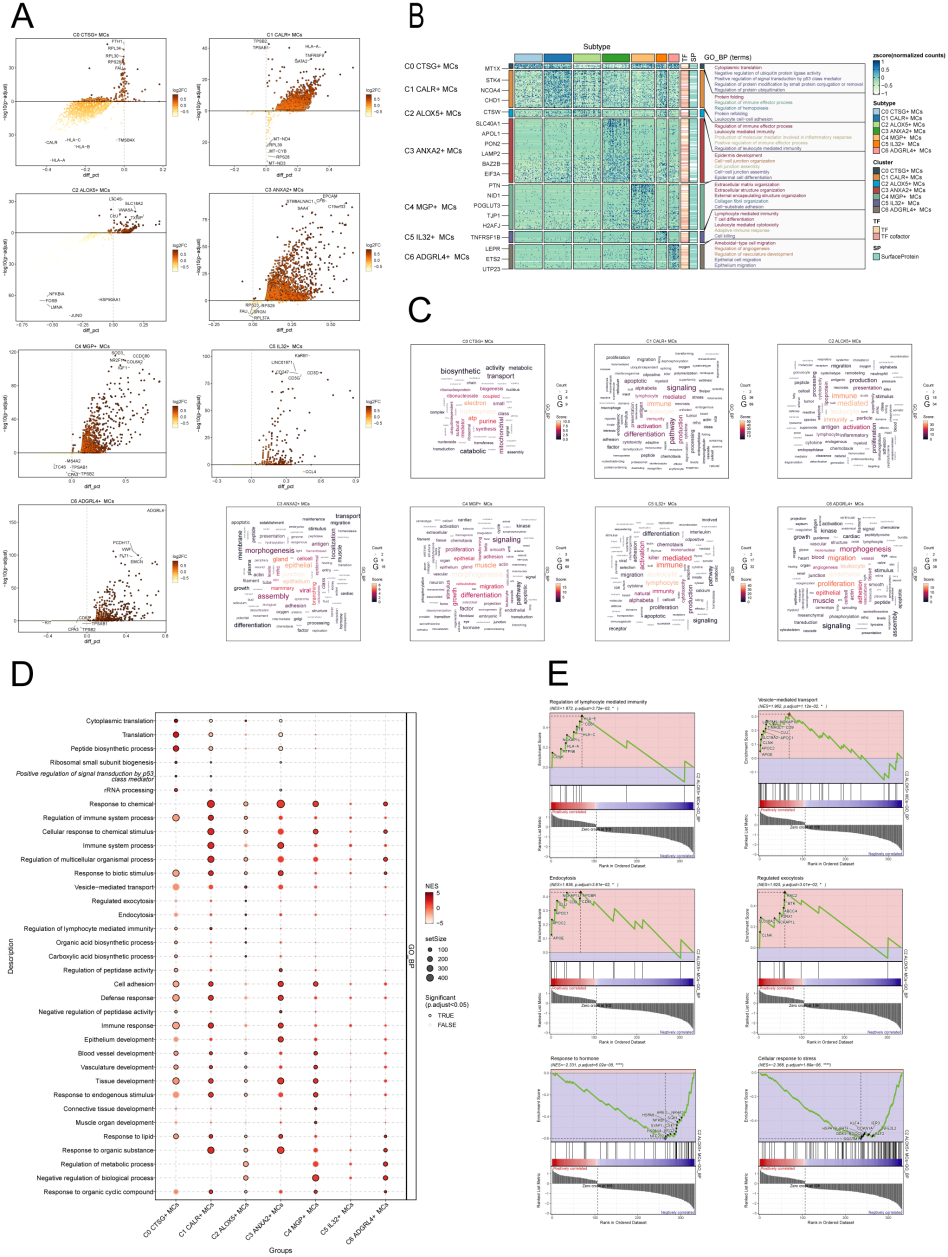
Enrichment analysis of MCs subpopulations in CC. **(A)** The volcanic plots provided descriptions of DEGs within each subgroup. **(B)** Heatmap showed the top5 enriched entries of GO-BP enrichment analysis for seven MCs subpopulations of differential genes. **(C)** Word cloud diagrams showed the results of GO-BP pathway in seven MCs subpopulations. **(D)** Based on the GO-BP entries, the results of enrichment analysis of differential genes in subpopulations of MCs were visualized using a bubble plot through GSEA. The size of the bubbles represents the number of genes enriched, while the color indicates the significance level. **(E)** The results of GSEA were presented, based on the GO-BP entries, showcasing the enriched pathways associated with differential genes in the C2 subpopulation of MCs.

First, the differential gene expression patterns among the MCs subgroups were shown using volcano plots in **Figure 5A**.

To further demonstrate the enrichment of DEGs in biological processes, we performed GO-BP enrichment analysis on DEGs in the MCs subpopulations. **Figure 5B** displayed the top five enrichment entries for different MCs subgroups, revealing unique pathways of enrichment among the seven subgroups. The results demonstrated distinct enrichment pathways among the seven MCs subgroups. The C0 CTSG+ MCs subgroup was primarily associated with pathways such as cytoplasmic translation, negative regulation of ubiquitin protein ligase activity, positive regulation of signal transduction by p53 class mediator. The C1 CALR+ MCs subgroup was enriched in pathways such as protein folding, regulation of immune effector process. The enrichment analysis conducted on the C2 ALOX5+ MCs subgroup revealed their close association with immune and inflammatory processes, including leukocyte mediated immunity, production of molecular mediator involved in inflammatory response, positive regulation of immune effector process. The C3 ANXA2+ MCs subgroup showed enrichment in pathways such as epidermis development, cell-cell junction organization. The C4 MGP+ MCs subgroup was enriched in pathways related to extracellular matrix organization, collagen fibril organization, and cell-substrate adhesion. The C5 IL32+ MCs subgroup mainly exhibited enrichment in pathways such as lymphocyte mediated immunity, leukocyte mediated cytotoxicity. The enrichment analysis of the C6 ADGRL4+ MCs subgroup revealed pathways related to regulation of angiogenesis, regulation of vasculature development, epithelial cell migration. Word cloud plots illustrate the enrichment results of DEGs in different pathways for the seven MCs subpopulations (**Figure 5C**). Additionally, the GSEA enrichment analysis results were visualized as bubble plots (**Figure 5D**).

Lastly, we conducted GSEA on the DEGs in the C2 subgroup of MCs, utilizing GO-BP terms. The results were depicted in **Figure 5E**. It was observed that the pathways associated with Regulation of lymphocyte-mediated immunity, Vesicle-mediated transport, Endocytosis and Regulated exocytosis were upregulated in the C2 subgroup. In contrast, the C2 subgroup exhibited downregulation of the pathways associated with the cellular response to stress and the response to hormone.

### CellChat analysis among cell subtypes

**Figure 6:**
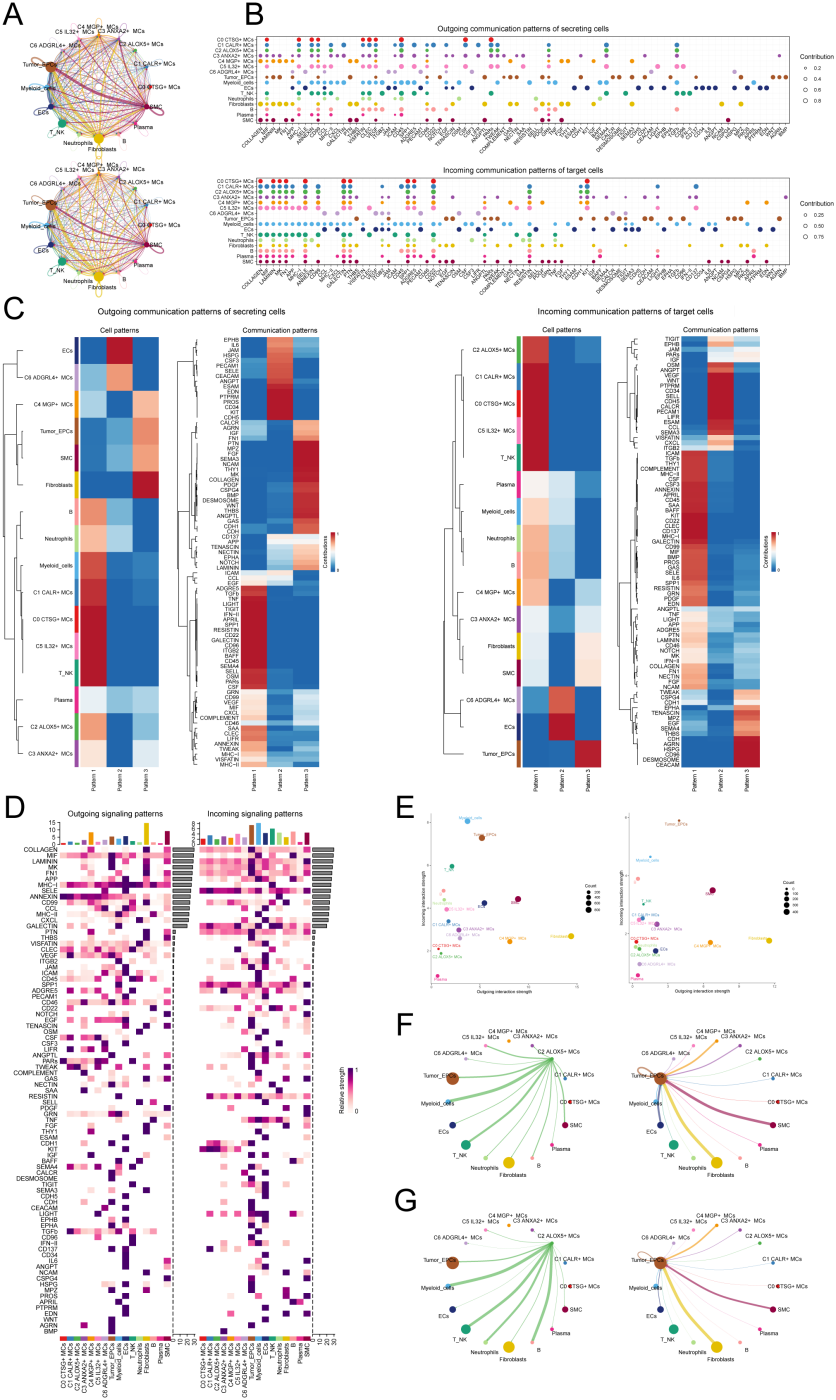
Presentation of CellChat results. **(A)** Circle plots depicted the number (top) and strength (bottom) of interactions among all cells in CC. **(B)** Dot plots showed the comparison of outgoing signaling patterns of secreting cells and incoming communication patterns of target cells. Higher contribution score implies the signaling pathway is more enriched in the corresponding cell group. **(C)** Heatmaps showed the outgoing communication patterns of secreting cells and incoming communication patterns of target cells, showing the correspondence between the inferred latent patterns and cell groups, as well as signaling pathways. **(D)** Heatmaps showed outgoing and incoming signal strength of all cell interactions in CC. **(E)** The scatter plot depicted the communication network analysis between all cells and the C2 subpopulation associated with tumor-related pathways, the color of the dots indicates different cells and the size of the dots indicates the number of cells. **(F, G)** Screening of the number **(F)** and strength **(G)** of cellular interactions circled plots with C2 ALOX5+ MCs as source and tumor as target.

To enhance our comprehension of the communication network among various cell types, decipher the intricacy of intercellular signaling, and investigate the functional and regulatory roles of cell subpopulations and crucial signaling pathways in physiological and disease processes, we utilized CellChat for the analysis and depiction of intercellular communication. Firstly, we constructed a communication network among all CC cells, including T_NK cells, ECs, fibroblasts, SMCs, EPCs, B cells, plasma cells, MCs, neutrophils, and myeloid cells. To determine the extent of cellular communication, we measured the quantity and intensity of these intercellular connections (shown by the thickness of the connecting lines). Higher numbers of intercellular contacts and higher levels of communication intensity are shown by thicker lines (**Figure 6A**). To examine the coordination and interaction among several cell subtypes, we employed CellChat to detect overall communication patterns and important signaling components inside various cell clusters. This allowed us to establish connections among cell populations. As a result, we discovered three outgoing signal patterns (viewing cells as senders) and three incoming signal patterns (viewing cells as receivers) (**Figure 6C**). The results revealed that most outgoing signals from MCs were dominated by pattern 1, involving multiple signaling pathways such as TIGIT, TNF, SPP1, and TWEAK. The incoming signals from all tumor cells exhibited pattern 3, including but not limited to CD96, CEACAM, and AGRN signaling pathways.

Next, to identify the key incoming and outgoing signals associated with the C2 ALOX5+MCs subpopulation and other cell subpopulations, we also identified receptor-ligand signaling related to the communication pathways (**Figures 6B, D**). The results showed that as secretory cells, the ligands associated with the output of C2 ALOX5+MCs were mainly MIF, CD45, and TWEAK. Regarding the input pathways in target cells, the receptors associated with C2 ALOX5+MCs were primarily CD99, SELE, and SPP1, while the receptors related to tumor cells included TWEAK, CEACAM, and CD96. **Figure 6E** displayed a scatter plot that showcased the communication network analysis of pathways associated with tumor interaction, both in all cells and specifically within the C2 subgroup of MCs.

**Figure 7:**
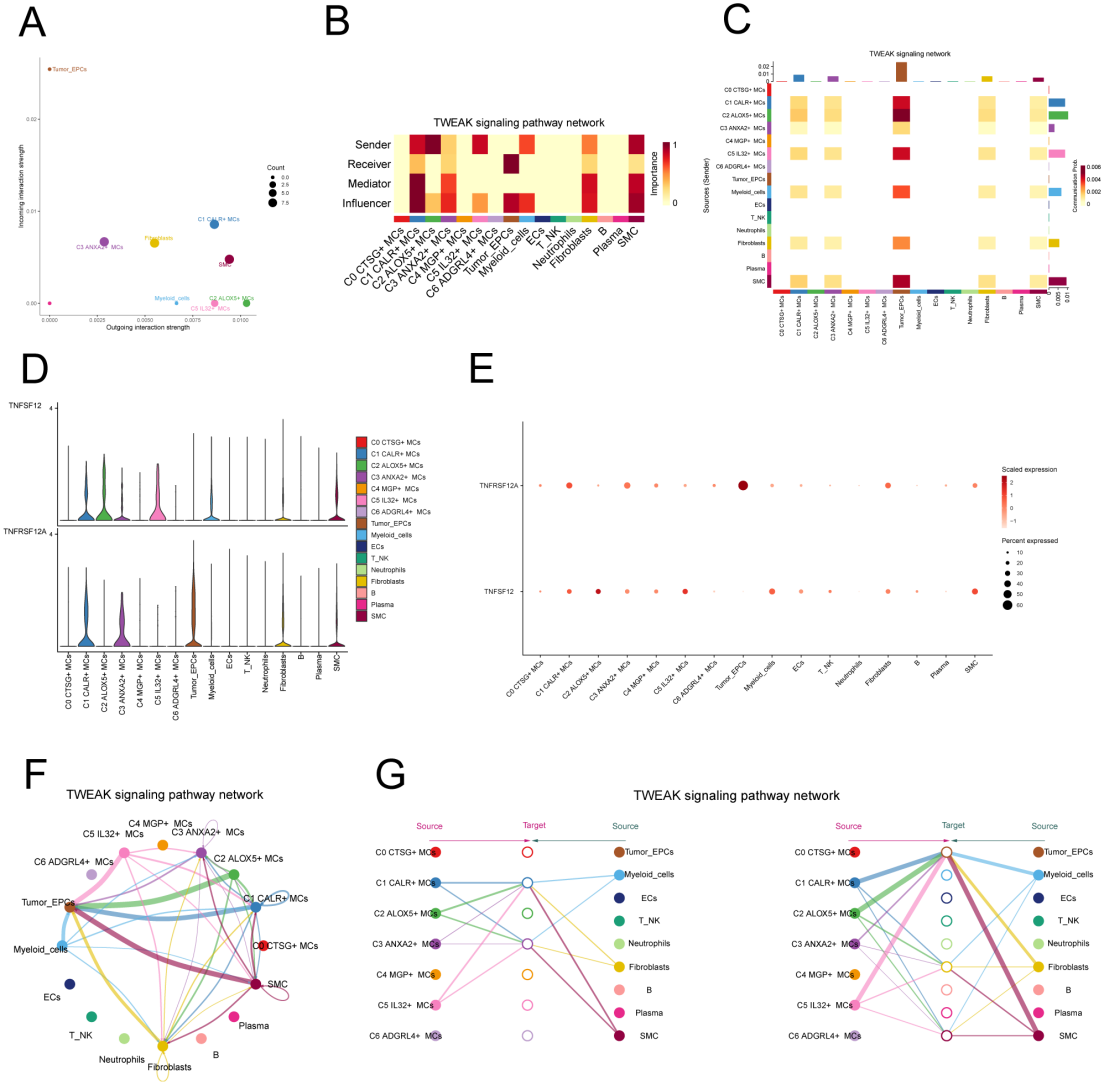
TWEAK signaling pathway. **(A)** Scatter plot of cellular communication patterns of TWEAK signaling pathway. The color of the dots indicates different cells and the size of the dots indicates the number of cells. **(B)** Heatmap showed the relative importance of each cell group based on the computed four network centrality measures of TWEAK signaling network. **(C)** Heatmap showed the centrality scores of TWEAK signaling pathways. **(D, E)** Violin and bubble plots demonstrated cellular interactions in the TWEAK signaling pathway. **(F, G)** Circle plot and hierarchical plot showed the inferred intercellular communication network for TWEAK signaling. The size of the circle is proportional to the number of cells in each cell group, and the edge width indicates the communication probability.

In addition, we chose C2 ALOX5+MCs as the source and tumor cells as the targets to study the interactions between MCs and tumor cells. The circular plot displayed the number (**Figure 6F**) and strength (**Figure 6G**) of cell-cell interactions between C2 ALOX5+MCs as the source and tumor cells as the targets. Combining the results from CellChat analyses, we found that the TWEAK signaling pathway exhibited strong interaction between ligands and receptors. The scatter plot revealed the cell-cell communication patterns of the TWEAK signaling pathway, emphasizing the significance of C2 CRYAB+MCs in this route (**Figure 7A**). Using the network centrality analysis of the TWEAK signaling network, we determined that the C2 MCs subpopulation had the highest level of importance as a sender in the TWEAK signaling pathway. Conversely, tumor cells were identified as the most significant receivers (**Figures 7B, C**). Significantly, the ligand-receptor pair TNFSF12 - TNFRSF12A was identified as a key element in the TWEAK communication network (**Figures 7D, E**). The circular and layered diagrams depicted the deduced network of cell-cell communication in TWEAK signaling (**Figures 7F, G**). All cell types identified in the CC tissue were examined as potential source cells for the TWEAK signaling pathway. The findings revealed that C1 CALR+MCs, C2 ALOX5+MCs, C3 ANXA2+MCs, C5 IL32+MCs, myeloid cells, fibroblasts, and SMCs are capable of targeting tumor cells by releasing TWEAK.

### *In vitro* experimental validation of TNFSF12A

**Figure 8:**
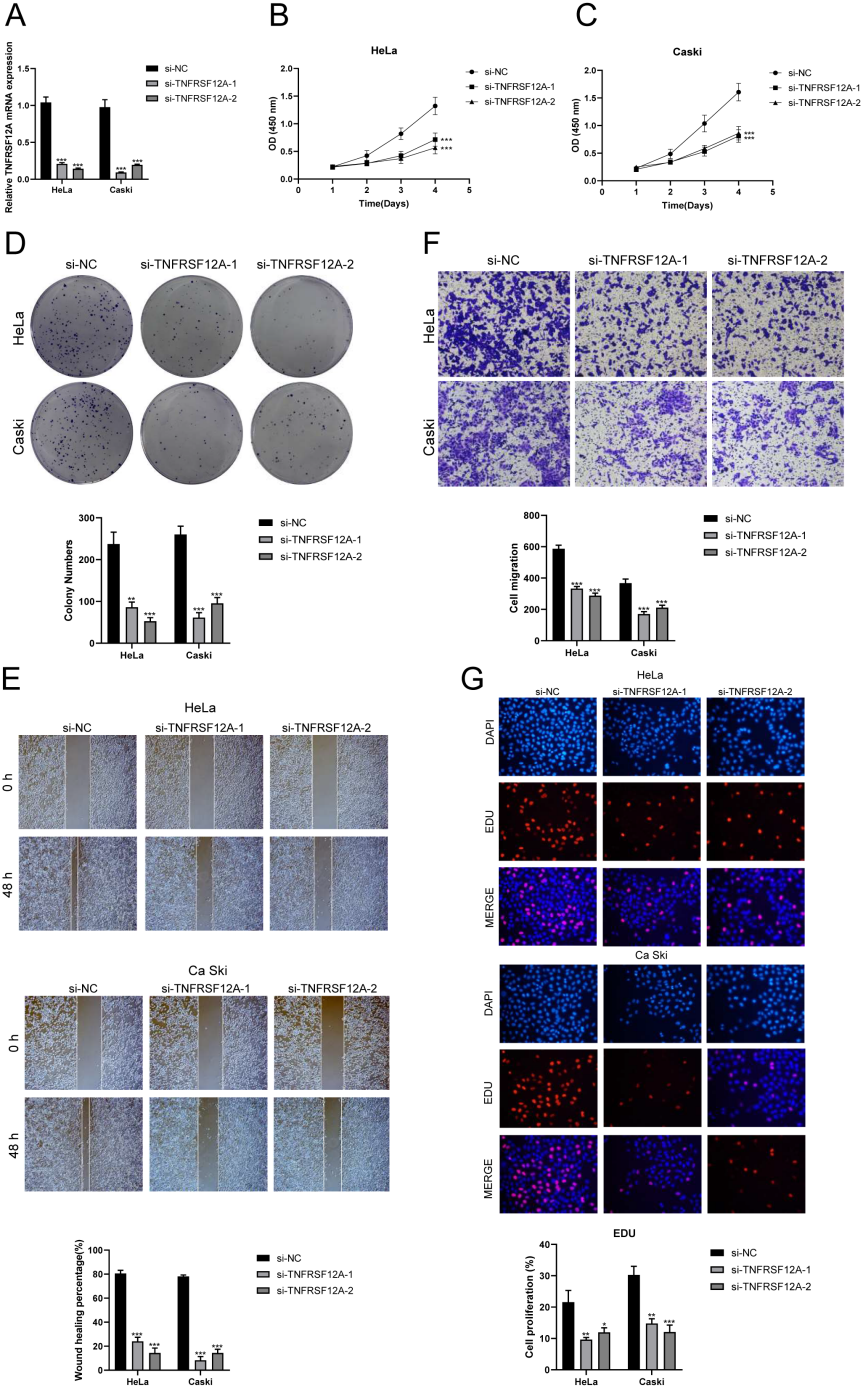
*In vitro* experimental validation of TNFSF12A. **(A)** The bar graph depicted the initial mRNA expression levels in Hela and Caski cell lines. **(B, C)** Cell viability was significantly diminished following the knockdown of TNFSF12A, as demonstrated by the CCK-8 assay. **(D)** The colony formation assay revealed that the number of colonies in cells with TNFSF12A knocked out was substantially lower compared to the si-NC group. **(E)** Scratch assays indicated that the knockdown of TNFSF12A markedly slowed the migration of Hela and Caski cells. **(F)** Transwell assays showed that the knockdown of the TNFSF12A gene significantly reduced the invasiveness of Hela and Caski cells. **(G)** EdU staining results suggested that the knockdown of the TNFSF12A gene inhibited the proliferation of Hela and Caski cells. *P< 0.05, **P< 0.01, and ***P< 0.001

TNFRSF12A, also known as Tumor Necrosis Factor Receptor Superfamily Member 12A, was a part of the TNFR superfamily. It had a diverse function in controlling cellular growth, viability, migration and apoptosis (69–71). Recent research has highlighted the significant impact of TNFRSF12A on the development, advancement, and metastasis of different types of cancer in humans (72, 73). Nevertheless, the exact function of TNFRSF12A in CC had yet to be clarified. To this end, we conducted *in vitro* functional assays to determine the impact of TNFRSF12A on CC cells. For precision and consistency, we performed all tests on two CC cell lines (HeLa and CaSki). Initially, we assessed the baseline mRNA expression levels in these cell lines (**Figure 8A**). Knocking down TNFRSF12A in these cell lines resulted in a notable reduction in the viability of tumor cells, as seen by the CCK-8 test (**Figures 8B, C**). Moreover, a substantial reduction in cellular proliferation was confirmed by colony formation and EdU assays following the TNFRSF12A knockdown in both cell lines (**Figures 8D, G**). These results indicated that the silencing of TNFRSF12A reduced tumor cell activity and proliferation, thereby impeding tumor growth. Furthermore, scratch and Transwell assays demonstrated a significant decrease in the migratory and invasive abilities of the TNFRSF12A-knockdown tumor cells in contrast to the control group (**Figures 8E, F**). These investigations collectively affirmed the critical significance of the TNFRSF12A gene regulatory network in the etiology and metastatic capacity of CC.

### Construction of a prognostic model associated with C2 ALOX5+ MCs score

**Figure 9:**
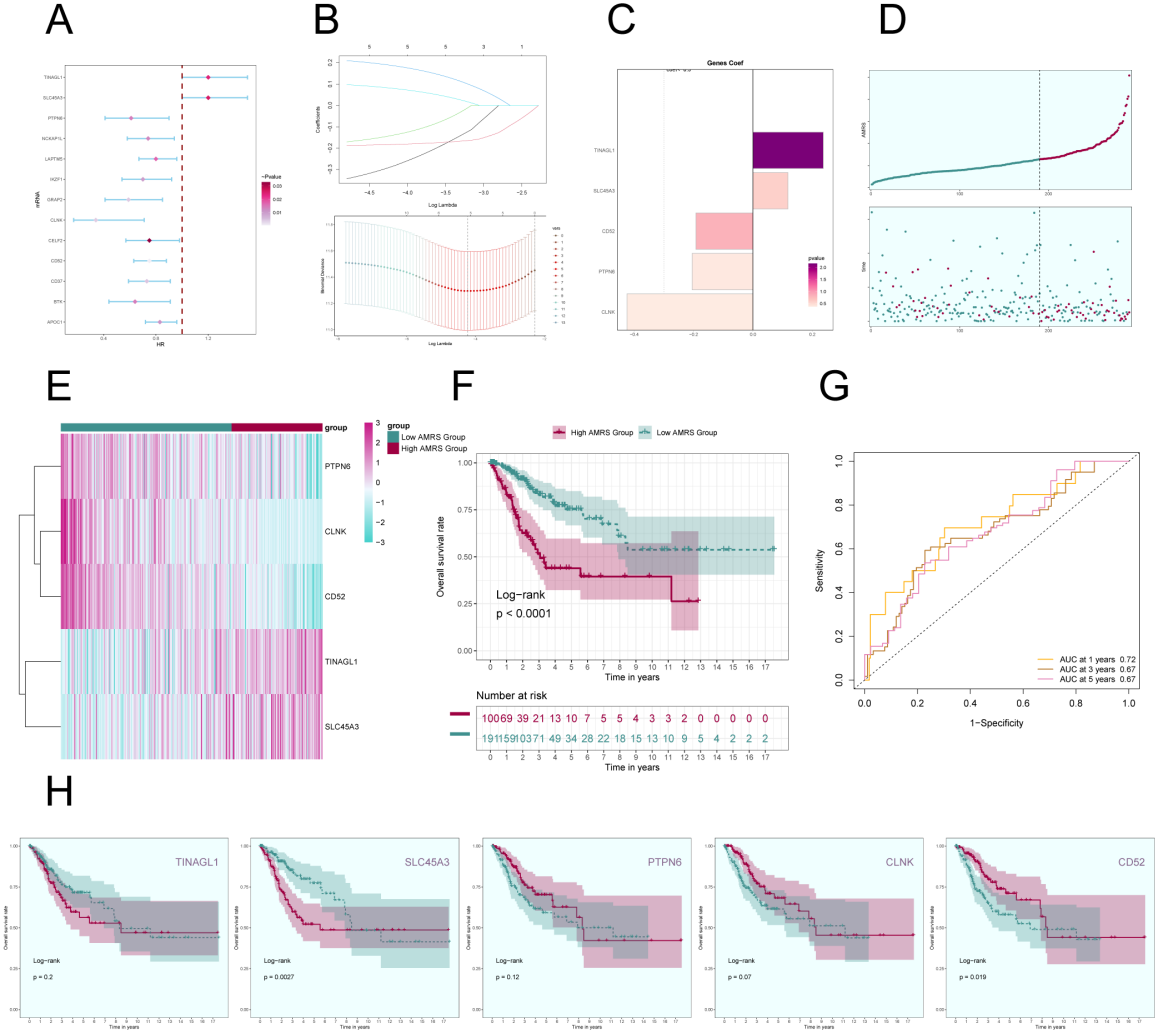
Construction of a prognostic model associated with C2 ALOX5+ MCs score. **(A)** Forest plot from univariate Cox regression analysis can be used to illustrate genes with statistically significant differences (*P*<0.05) (HR<1: protective factor, HR>1: risk factor). **(B)** Through LASSO regression analysis, five genes (non-zero regression coefficients) associated with prognosis were selected. The optimal parameter (lambda) was determined through ten-fold cross-validation (above), and the LASSO coefficient curve was determined by the optimal lambda (below). **(C)** Bar graph displayed the Coef values of the genes utilized for model construction. **(D)** Curve plots showed risk scores for the high AMRS group and the low AMRS group (top), and scatter plots showed survival status of both groups over time for survival/death events (bottom). AMRS: ALOX5+ MCs Risk Score. **(E)** Heatmap showed differential expression of modeled genes, with color scales based on normalized data. **(F)** Kaplan-Meier curves showed the survival difference between the high AMRS group and the low AMRS group. **(G)** The sensitivity and specificity of 1, 3, and 5-year outcomes were assessed through ROC curve and AUC values. **(H)** The Kaplan-Meier curves individually demonstrated the differences in survival among patients grouped based on the expression levels of five prognostic-related genes (TINAGL1, SLC45A3, PTPN6, CLNK, CD52).

In order to gain a deeper understanding of the key significance of MCs with high expression of ALOX5 in the prognosis of CC, and to offer more precise recommendations for clinical practice, we have created a risk scoring model. First, a univariate Cox regression analysis was conducted to identify the top 100 DEGs that are linked to C2 ALOX5+MCs. The study revealed 13 genes that had a significant correlation with prognosis (*P*<0.05) (**Figure 9A**). TINAGL1 and SLC5A3 exhibited a hazard ratio (HR > 1), signifying that these two genes are prognostic risk factors, whereas the other genes functioned as protective factors. To address the problem of multicollinearity among these genes, we conducted further selection using LASSO regression analysis. This led to the discovery of five genes that were shown to be linked with prognosis, as shown in **Figure 9B**. Afterwards, the coefficient values were computed using multivariable Cox regression analysis (**Figure 9C**).

Afterwards, using the expression levels and regression coefficients of the five chosen prognostic-related genes, we computed the ALOX5+MCs score for each patient using the following formula: ALOX5+MCs score = (0.24) × (TINAGL expression level) + (0.12) × (SLC45A3 expression level) + (-0.19) × (CD52 expression level) + (-0.21) × (PTPN6 expression level) + (-0.42) × (CLNK expression level). The ALOX5+MCs risk score (AMRS) was utilized to classify the participants into high-risk and low-risk groups, based on the optimal cutoff value. The curve plot and scatter plot illustrated the disparities in risk scores and survival rates between the two groups (**Figure 9D**), suggesting a link between higher AMRS and unfavorable prognosis. Furthermore, a heatmap illustrated the distinct patterns of gene expression employed in constructing the model (**Figure 9E**).

**Figure 9F** displayed the Kaplan-Meier curve showing the contrast in survival rates between the high AMRS group and the low AMRS group, supporting the conclusion of worse survival outcomes in the high AMRS group. The model’s predictive accuracy was assessed by examining its sensitivity and specificity over 1, 3, and 5 years with ROC curves and AUC values (**Figure 9G**). The results indicated that the model had predictive value. Finally, survival analysis was performed on the five prognostic-related genes (TINAGL1, SLC45A3, PTPN6, CLNK, CD52) used in the model (**Figure 9H**), further confirming that SLC45A3 was risk factors associated with poorer prognosis in the high AMRS group, while CD52 was protective factors associated with better prognosis in the high AMRS group.

### Nomogram construction and correlation analysis of risk scores and modeled genes

**Figure 10:**
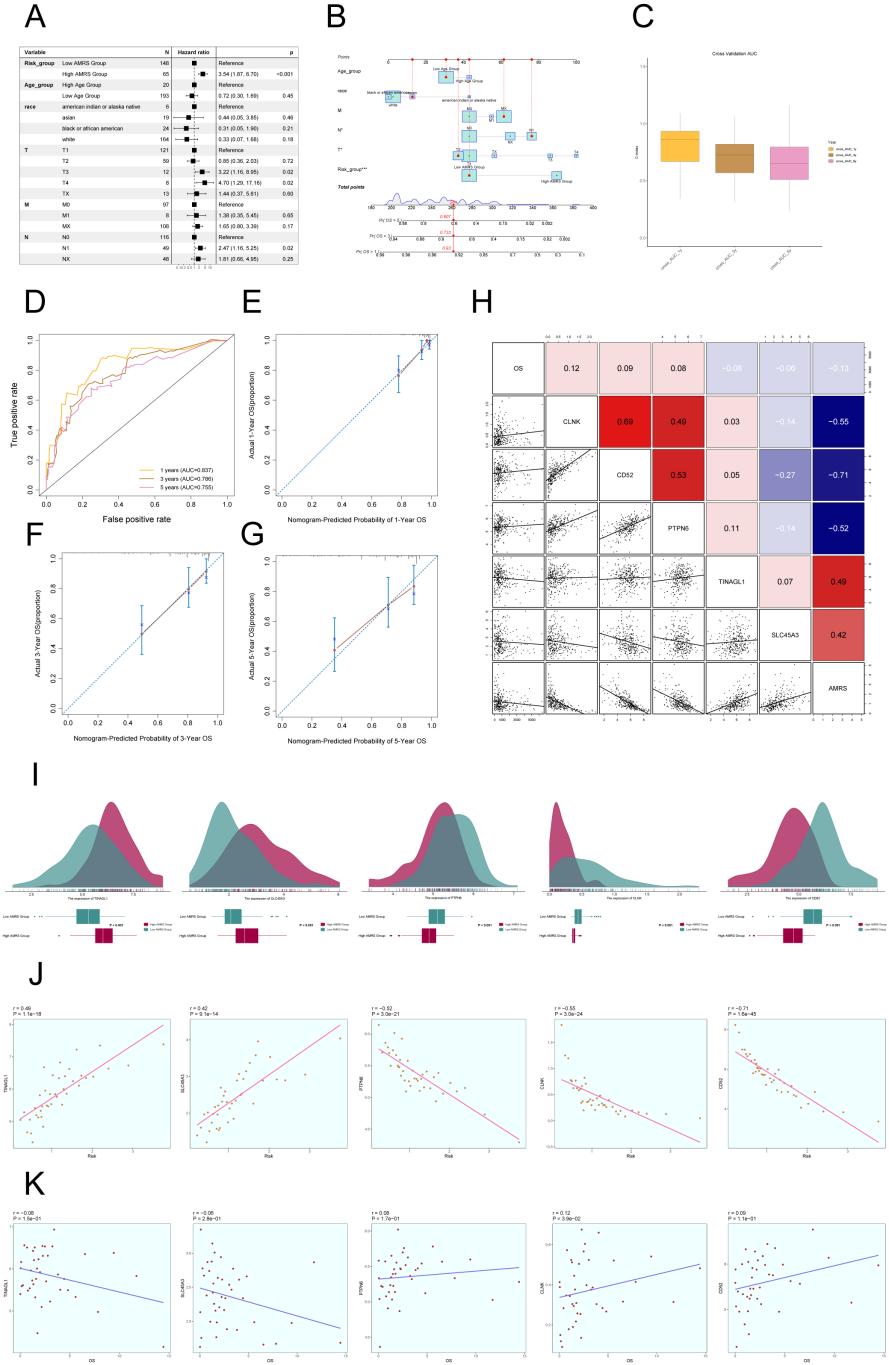
Construction of Risk Score Model for C2 ALOX5+MCs. **(A)** The Forest plot demonstrated the results of Multivariate Cox regression analysis integrating risk scores and clinical factors (age, race and tumor clinical stage T, M and N). **(B)** Nomogram showed the prediction of 1, 3, and 5-year of OS based on race, tumor clinical stage (T, M, and N), age, and risk score, with the most significant difference in the risk score group. **(C)** The box-line plot displayed visualizations of the C-index for cross-validation at 1, 3, and 5 years. **(D)** ROC curves showed nomogram AUC at 1,3,5 years. **(E–G)** Calibration curves validated the accuracy of the nomogram in predicting the 1-year, 3-year, and 5-year survival rates. **(H)** Heatmap and Scatter plots demonstrated the correlation between prognostic genes, OS, and genes used in model establishment. **(I)** Ridge and box plots showed the expression differences of prognosis-related genes in the high AMRS group and low AMRS group. High and low peaks indicate the patient density of patients with this gene expression. **(J)** The scatter plot illustrated the correlation between the risk scores and the genes utilized for model construction. **(K)** Scatter plot displayed the correlation analysis between the constructed model genes and the OS. *P < 0.05, and ***P < 0.001.

In order to confirm the autonomy of the AMRS as a predictive factor, we performed a multivariable Cox regression analysis that included the risk score along with clinical variables such as age, race, and tumor stages T, M, and N. The findings indicated that AMRS independently impacts patient prognosis as a risk factor (*P*< 0.05) (**Figure 10A**). To improve the accuracy of predicting patient survival rates, a nomogram was created using factors such as race, tumor stage, age, and risk score to forecast the likelihood of survival at 1, 3, and 5 years. The results indicated that the differences were most significant in the AMRS group (**Figure 10B**). Additionally, the Nomogram’s prediction was confirmed by evaluating the C-index and ROC curves. The obtained AUC values were 0.837 (1 year), 0.786 (3 years), and 0.755 (5 years), confirming the accuracy of the model (**Figures 10C, D**). Similarly, the calibration curves demonstrated that the nomogram effectively predicted actual survival outcomes (**Figures 10E–G**). In addition, scatter plots were employed to examine the relationship between the five genes included in the model and the ALOX5+MCs score and OS (**Figure 10H**), as well as the variations in gene expression between the high AMRS and low AMRS groups (**Figure 10I**). Finally, the correlation analysis showed a positive association between TINAGL1, SLC45A3, and the risk score, and a negative correlation with OS. On the other hand, PTPN6, CLNK, and CD52 demonstrated a negative correlation with the risk score, and a positive correlation with OS. These findings were illustrated in **Figures 10J, K**.

### Comparative examination of immune infiltration in groups with high and low scores of ALOX5+ MCs

**Figure 11:**
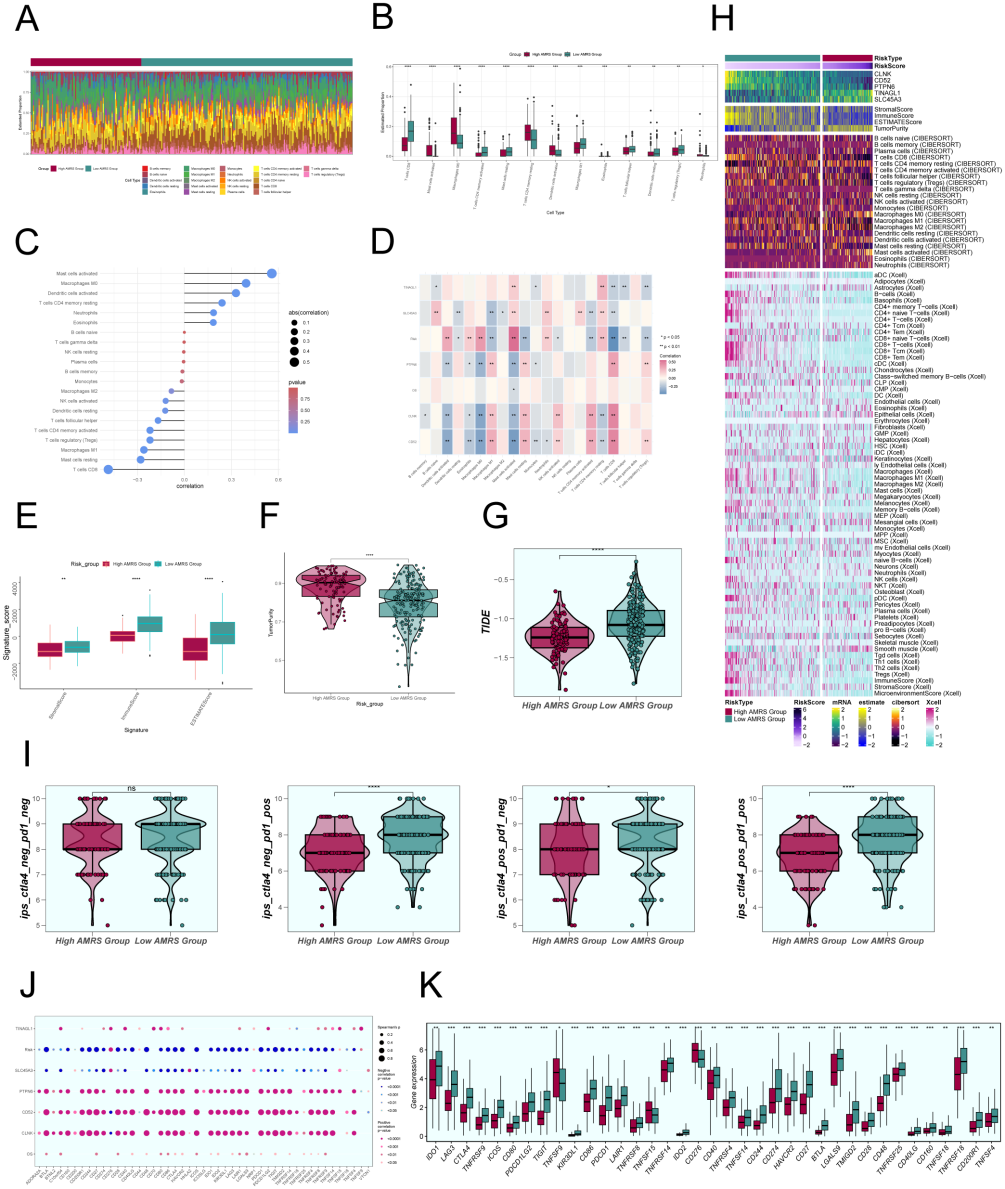
Comparative Analysis of Immune Infiltration between High and Low AMRS Groups. **(A)** Stacked bar graph illustrated the distribution of 22 immune cell types between the high and low AMRS groups. **(B)** Boxplots showed the estimated proportion of 13 immune cells types between the high AMRS group and the low AMRS group of CC patients. **(C)** Bar graph showed correlation between immune cells and risk scores. **(D)** Heatmap showed correlation analysis between immune cells and construct model genes, OS, and risk scores. **(E)** The differences in stromal score, immune score, and ESTIMATE score between the high and low AMRS groups of CC patients. **(F)** Boxplots showed the level of tumor purity between the high AMRS group and low AMRS group. *P<0.05, **P<0.01, ***P<0.001, and ****P< 0.0001 indicated a significant difference. **(G)** The differences in the levels of TIDE between the high and low AMRS groups. **(H)** Heatmap showed the difference in modeling genes, StromalScore, ImmuneScore, ESTIMATScore, TumorPurity, and the level of immune cell infiltration calculated using CIBERSORT, Xcell between the high and low AMRS groups. Color scales are based on standardized data. **(I)** Boxplots compared the sensitivity of two immunotherapeutic drugs, ctla4 and pd1, in the high and low AMRS groups. **(J)** Bubble plots showed correlations between modeled genes, risk scores, OS, and immune checkpoint-related genes. **(K)** Boxplots showed the expression levels of immune checkpoint-related genes in the high AMRS group and low AMRS group. Red: high AMRS group; Green: low AMRS group.

To investigate the differences in immune cell composition across varying risk score categories of AMRS, we analyzed the presence of 22 immune cell types in CC patients from the TCGC database using the CIBERSORT algorithm, as shown in **Figure 11A**. **Figure 11B** displayed the percentages of 13 immune cell categories that showed variances between the two groups in box plots. The results indicated that the High AMRS group had a higher proportion of Macrophages M0, MCs activated, T cells CD4 memory resting, and Dendritic cells activated, while the Low AMRS group had higher proportions of T cells CD8, T cells CD4 activated, MCs resting, and Macrophages M1.

Subsequently, we evaluated the correlation between immune cells and AMRS, as shown in **Figure 11C**. The results demonstrated a significant positive correlation between AMRS and MCs activated, Macrophages M0, and a negative correlation with T cells CD8, MCs resting, among others. The heatmap visualized the correlation analysis between immune cells, the modeled genes, OS, and the risk score (**Figure 11D**), with results displayed in the figure. We further observed differences in the StromalScore, ImmuneScore, and ESTIMATEScore, as well as tumor purity between the High AMRS group and the Low AMRS group (**Figure 11E**). In particular, the High AMRS group exhibited lower scores across all three measures when compared to the Low AMRS group. The visualization of tumor purity (**Figure 11F**) indicated that the High AMRS group had higher tumor purity values than the Low AMRS group. The TIDE values between the two groups also exhibited differences (**Figure 11G**).

The heatmap displayed in **Figure 11H** illustrated the differences in modeled genes, StromalScore, ImmuneScore, ESTIMATEScore, TumorPurity, and immune cell infiltration levels between the High AMRS group and the Low AMRS group, as calculated using CIBERSORT and Xcell algorithms. Furthermore, we compared the sensitivity of two immunotherapeutic drugs, CTLA4 and PD1, in the High AMRS group and the Low AMRS group using box plots (**Figure 11I**). The results showed that the sensitivity levels were generally lower in the High AMRS group compared to the Low AMRS group, particularly in the groups of CTLA4-neg/PD1-pos and CTLA4-pos/PD1-pos, with significant differences observed. The bubble plot (**Figure 11J**) displays the correlation between immune checkpoint-related genes and the modeled genes, risk score, and OS. It indicates a strong positive correlation between PTPN6, CD52, CLNK, and most immune checkpoints, while SLC45A3 demonstrated a negative correlation with most immune checkpoints. Finally, the expression levels of immune checkpoint-related genes were analyzed, indicating higher expression in the majority of immune checkpoint-related genes in the Low AMRS group compared to the High AMRS group (**Figure 11K**).

### Enrichment analysis

**Figure 12:**
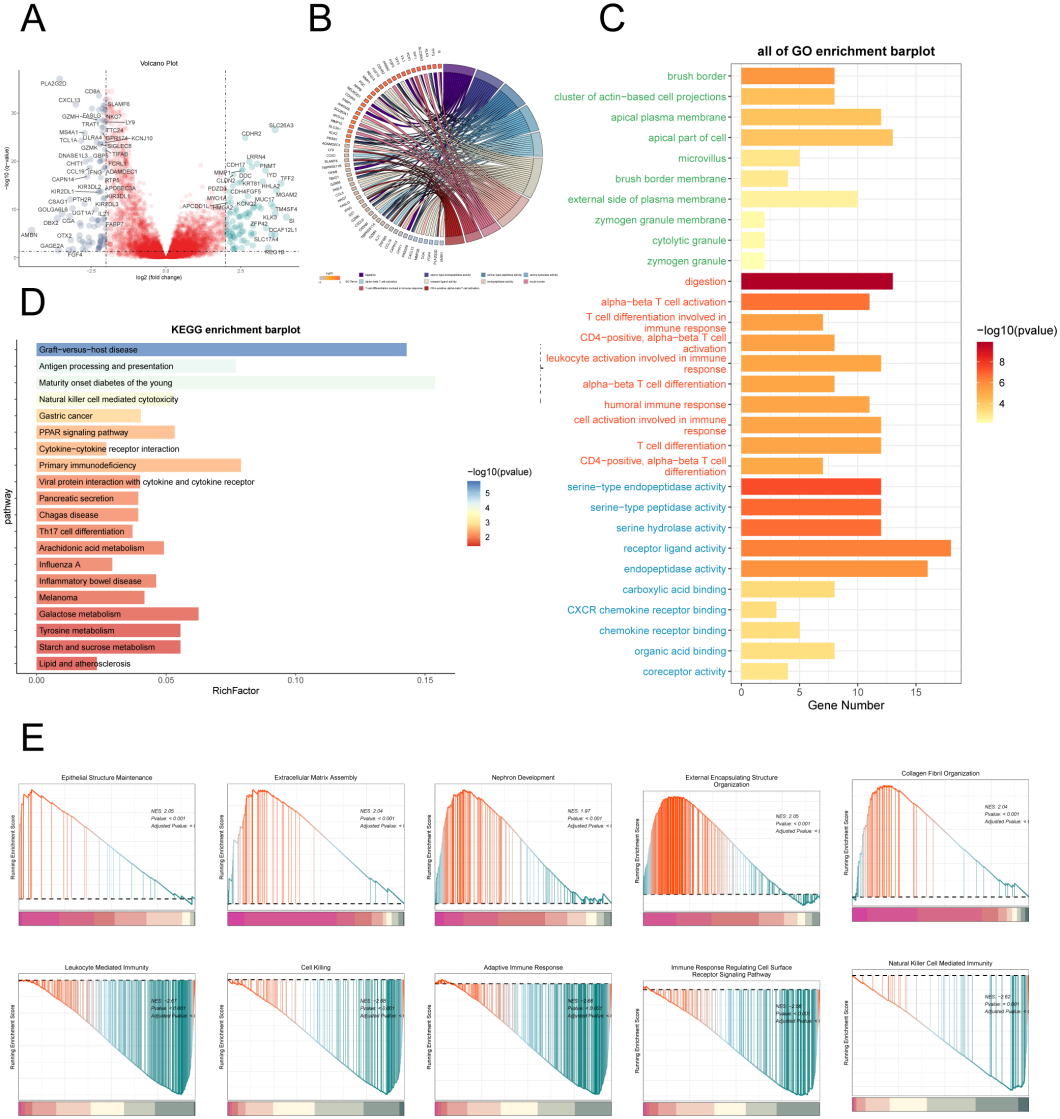
Enrichment analysis. **(A)** Volcano plot depicted differential gene distribution between the high AMRS group and low AMRS group. **(B, C)** Chord and bar graphs showed the results of GO Enrichment Analysis of differential genes in the high AMRS group and low AMRS group. **(D)** Bar graph showed the results of KEGG enrichment analysis of DEGs. **(E)** The GSEA was conducted to analyze the results of DEGs in the high and low AMRS groups.

To delve deeper into the differences between the High AMRS group and the Low AMRS group, we analyzed DEGs and showcased the expression patterns of these unique genes through a volcano plot (**Figure 12A**). Afterwards, in order to obtain a more thorough comprehension of the biological importance and operational traits of the distinct genes, we utilized different enrichment techniques to examine the DEGs in each group. The results of the GO analysis unveiled a noteworthy enrichment of differential gene expression in pathways such as digestion, serine-type endopeptidase activity, serine hydrolase activity, among others (**Figure 12C**). The genes associated with these enriched terms are depicted in **Figure 12B**. Furthermore, the outcomes of the KEGG enrichment analysis were visually presented using a bar graph, affirming the significant associations between these differential genes and pathways such as Graft-versus-host disease, Antigen processing and presentation, Maturity onset diabetes of the young, and Natural killer cell mediated cytotoxicity (**Figure 12D**). Moreover, utilizing the enriched GO-BP terms as a basis, GSEA was conducted, and the results are illustrated in **Figure 12E**.

## Discussion

Single-cell sequencing, as an emerging technology, has exhibited its unique advantage in uncovering tumor heterogeneity (74). In recent years, multiple studies utilized this technology to analyze various solid tumors, providing optimized guidance for clinical diagnosis and treatment strategies (75–77). MCs, as innate immune cells, played a role in both tumor suppression and promotion, with the effects varying depending on the cancer type (11, 78). Currently, there is ongoing controversy regarding the impact of MCs on CC. Research has indicated that the risk scores derived from prognostic models for CC may correlate with the infiltration of immune cells such as MCs (79). Additionally, MCs may facilitate the invasion and metastasis of CC cells by releasing histamine and cannabinoids (80). However, the mechanisms underlying the role of MCs in CC remain unclear. Consequently, we have undertaken an extensive investigation into this area. In this study, we employed single-cell sequencing technology to demonstrate the microenvironment landscape of CC, confirming the existence of immune cells, EPCs, and MCs as distinct cellular subgroups. Moreover, we observed that the histotype of MCs within cervical carcinomas was predominantly H-group, which is commonly considered to be associated with an elevated risk of CC progression, leading us to surmise that MCs might be implicated in the progression of CC.

Subsequently, our analysis of MCs subgroups revealed a particular subset known as the C2 subgroup, characterized by significantly upregulated expression of arachidonate 5-lipoxygenase (ALOX5). ALOX5, a constituent of the lipoxygenase gene family, served a crucial function in both inflammation and malignancy. ALOX5 affected tumor occurrence and development through catalyzing the metabolism of arachidonic acid and was closely associated with poor prognosis in various malignant tumors (81–83). Enrichment analysis results revealed that subgroup C2 had a crucial impact on several biological processes, such as the regulation of immune effector processes, leukocyte-mediated immunity, the production of molecular mediators involved in inflammatory responses, the regulation of lymphocyte-mediated immunity, vesicle-mediated transport, and endocytosis. This demonstrated that subgroup C2 had a key role in immunity and inflammatory responses, and, according to previous studies, these biological processes were often closely associated with tumors (84–87). Moreover, our research discovered that the C2 subgroup had a higher proportion in the H and T-group compared to other subgroups, and relative to those subgroups, the C2 subgroup had a higher CNV score. Therefore, we hypothesized that the C2 subgroup possessed a higher degree of malignancy and may be correlated with the prognosis of CC.

Analysis results from Slingshot demonstrated that Lineage1 and Lineage2 represented the differentiation trajectories of tumor-associated MCs and normal cells, respectively, but their differentiation endpoints differed completely. The C2 subgroup was in the middle to late stages of both differentiation trajectories. We observed differences between the two trajectories after passing through the C2 subgroup, setting forth the hypothesis that the transformative effect of the C2 subgroup might be the reason for these differences. The C2 subgroup could potentially serve as a transformative MCs subset associated with tumor-related events, playing a pivotal role in the transition from benign to malignant states.

Considering the potential interactions between tumor cells and other cells, we conducted an analysis of intercellular communication involving the C2 subgroup. Research results demonstrated that the C2 subgroup interacted with tumor cells through the TWEAK signaling pathway. Its receptor, TNFSF12A, induced cell apoptosis and was associated with tumor cell migration and invasion (88, 89). To validate these findings, we conducted *in vitro* experiments on Hela and Caski cell lines, which revealed that the downregulation of TNFRSF12A suppressed CC tumor growth and migration, thereby confirming the critical role of the TNFRSF12A gene regulatory network in CC occurrence and metastatic potential and further supporting our hypotheses.

As controversies persist regarding the prognosis of CC patients in relation to MCs, we identified 13 genes associated with CC prognosis and constructed a risk scoring model. It is noteworthy that LASSO regression analysis identified five genes associated with prognosis, including TINAGL1 and SLC5A3 as risk factors, and CD52, PTPN6, and CLNK as protective factors. The coexistence of risk and protective factors among the prognosis-related genes led us to speculate that MCs of CC may induce the expression of these genes to promote tumor immune evasion and metastasis, exerting immunosuppressive effects. Protective genes were considered associated with a lower disease risk and generally indicative of a better prognosis. These findings suggest that the C2 subgroup may possess the potential to push the prognosis of CC towards either a poor or favorable outcome, serving as a crucial component in the transition between tumor malignancy and benign status, further validating our previous hypotheses. Utilizing external TCGA data, the prognostic significance of MCs infiltration was assessed, uncovering a link between elevated AMRS and reduced OS. Additionally, this finding was confirmed in a group of patients in a clinical setting.

Given the extensive presence of immune cells in the CC microenvironment, we conducted a comparison of this infiltration in different risk assessment categories. The high AMRS group showed elevated levels of immunosuppressive cells, as well as notable variations in matrix scores, immune scores, and ESTIMATE scores when compared to the low AMRS group. Our research indicated that individuals in the low AMRS category may have a higher chance of responding positively to anti-PD-1 treatment. It is worth mentioning that according to the immune checkpoint analysis results, we discovered that the TME of patients in the low AMRS group may contain a greater number of infiltrating T cells that express immune checkpoint-related proteins. Consequently, patients in this group may be more responsive to ICB therapy, whereas the high AMRS group may be resistant or unresponsive to ICB therapy, which is in accordance with our research. Further corroborating previous research, our findings support the conclusion that patients with advanced CC exhibit lower responsiveness to ICB therapy (6, 9). In summary, our comprehensive research findings suggest that C2 ALOX5+ MCs may be associated with the progression and malignant transformation of CC. Targeted studies on this subpopulation could potentially enhance the therapeutic efficacy for CC and pave the way for uncovering new therapeutic targets and mechanisms underlying the disease, thereby offering novel avenues for future intervention and treatment strategies.

## Conclusion

Building on the single-cell characteristics of CC, we investigated the heterogeneity within the TME of CC. Further analysis of MCs subgroups identified the distinct presence of the C2 ALOX5+MCs subgroup in CC, suggesting its potential role as a tumor-associated MCs subgroup with transformative effects on immunity and inflammation. Importantly, coupled with CellChat analysis, we discovered that TNFRSF12A may facilitate the growth and migration of CC, a finding corroborated by *in vitro* experiments. These findings may unveil the crucial roles of TNFRSF12A in CC diagnosis, prognosis, and immune function, indicating its potential as a promising predictive marker and therapeutic target in CC patients. Subsequently, we developed a prognostic model to predict the survival outcomes in CC patients and assessed immune infiltration in different risk groups, offering novel insights for patient prognosis and treatment guidance. However, despite the valuable insights provided by our analysis, the research was limited to a specific group of individuals for validation, which highlights the need for a larger and more diverse clinical sample as well as future prospective studies to ensure wider generalizability. In addition, our samples are derived from public databases, which may have inherent biases or limitations. It is crucial to acknowledge any potential biases associated with these choices and consider their impact on the generalizability of the research findings. Additionally, although our findings were validated *in vitro*, extrapolating these conclusions to the whole organism remains challenging, underscoring the need for *in vivo* experimentation. Finally, the prognostic models employed in our research necessitate refinement. We aim to gather more reliable data in the future to enable more comprehensive and precise investigations.

## Data Availability

The original contributions presented in the study are included in the article/**Supplementary Material**. Further inquiries can be directed to the corresponding authors.

## Supplementary material

Supplementary Figure 1 The analysis of CNV results. **(A)** Heatmap showed the inferCNV profiles of EPCs using ECs as a reference. The red color indicates copy number increase and the blue color indicates copy number decrease. **(B)** Heatmap showed the inferCNV for each MCs subpopulation using ECs as a reference. The red indicates copy number increase and the blue indicates copy number decrease.
