# Supplementary figures and images for: Elucidating the role of tumor-associated ALOX5+ mast cells with transformative function in cervical cancer progression via single-cell RNA sequencing
Source: Front Immunol. 2024 Aug 19;15:1434450. doi: 10.3389/fimmu.2024.1434450 (PMC11366577; doi:10.3389/fimmu.2024.1434450)

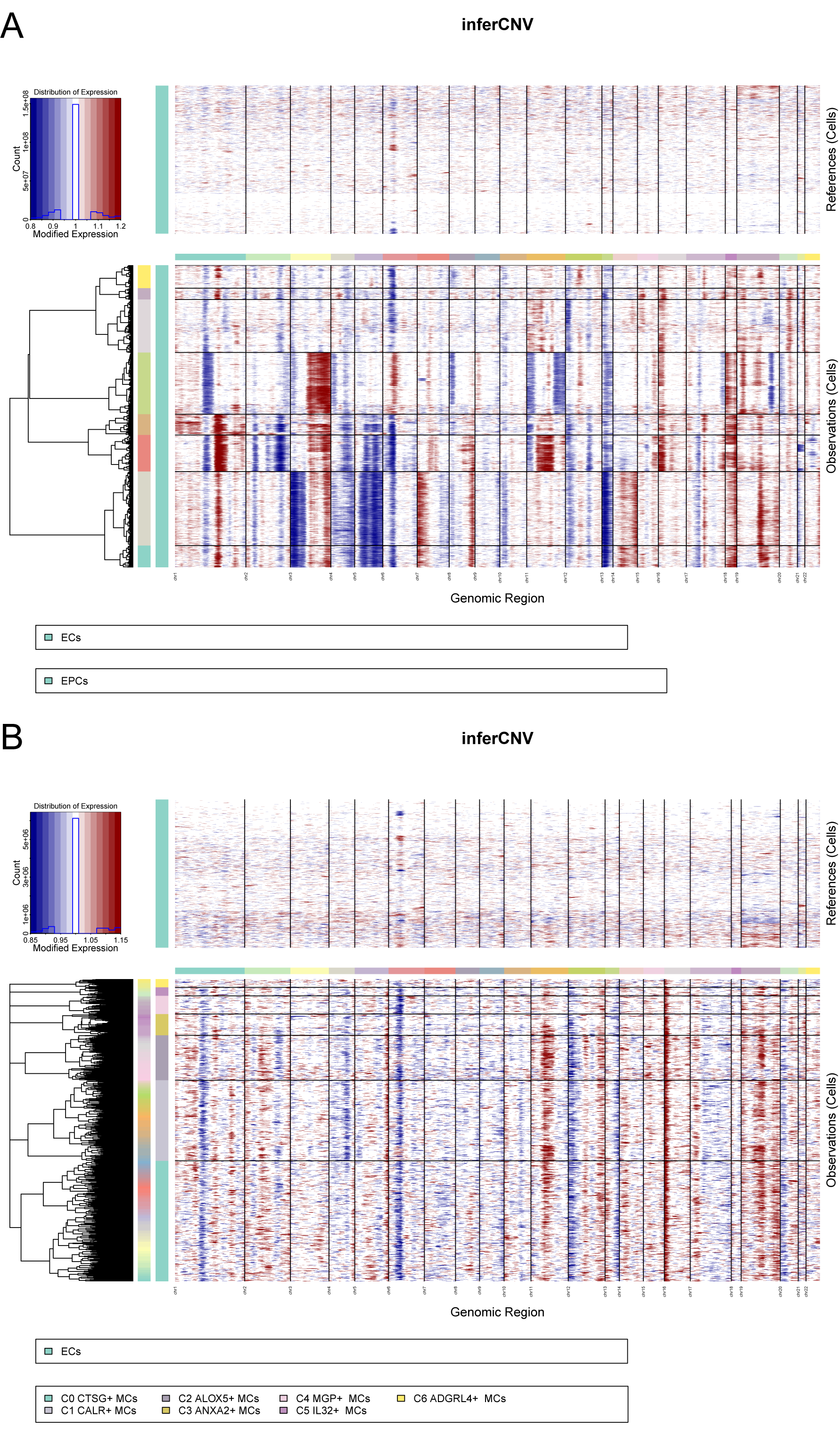

Supplement: Supplementary Figure 1 — The analysis of CNV results. (A) Heatmap showed the inferCNV profiles of EPCs using ECs as a reference. The red color indicates copy number increase and the blue color indicates copy number decrease. (B) Heatmap showed the inferCNV for each MCs subpopulation using ECs as a reference. The red indicates copy number increase and the blue indicates copy number decrease. [file Image1.tif]
